# An Enhanced Lightning Attachment Procedure Optimization with Quasi-Opposition-Based Learning and Dimensional Search Strategies

**DOI:** 10.1155/2019/1589303

**Published:** 2019-08-01

**Authors:** Tongyi Zheng, Weili Luo

**Affiliations:** School of Civil Engineering, Guangzhou University, Guangzhou, China

## Abstract

Lightning attachment procedure optimization (LAPO) is a new global optimization algorithm inspired by the attachment procedure of lightning in nature. However, similar to other metaheuristic algorithms, LAPO also has its own disadvantages. To obtain better global searching ability, an enhanced version of LAPO called ELAPO has been proposed in this paper. A quasi-opposition-based learning strategy is incorporated to improve both exploration and exploitation abilities by considering an estimate and its opposite simultaneously. Moreover, a dimensional search enhancement strategy is proposed to intensify the exploitation ability of the algorithm. 32 benchmark functions including unimodal, multimodal, and CEC 2014 functions are utilized to test the effectiveness of the proposed algorithm. Numerical results indicate that ELAPO can provide better or competitive performance compared with the basic LAPO and other five state-of-the-art optimization algorithms.

## 1. Introduction

Optimization problems can be found in many engineering application domains and scientific fields which have a complex and nonlinear nature. It is usually difficult to solve these optimization problems using classical mathematical methods since such methods are often inefficient and have a requirement of strong math assumptions. Due to the limitations of classical approaches, many natural-inspired stochastic optimization algorithms have been proposed to conduct global optimization problems in the last two decades. Such optimization algorithms were commonly simple and easy to implement, and these features make the possibility to solve highly complex optimization problems. These metaheuristics can be roughly classified into three categories: evolutionary algorithms, swarm intelligence, and physical-based algorithms.

Evolutionary algorithms are generic population-based metaheuristics, which imitate the evolutionary behavior of biology in nature such as reproduction, mutation, recombination, and selection. The first generation starts with randomly initialized solutions and further evolves over successive generations. The best individual among the whole population in the final evolution is considered to be the optimization solution. Some of the popular evolutionary algorithms are genetic algorithm (GA) [1], genetic programming (GP) [2], evolution strategy (ES) [3], differential evolution (DE) algorithm [4], and biogeography-based optimizer (BBO) [5].

Swarm intelligence algorithms mimic the collective behavior of swarms, herds, schools, or flocks of creatures in nature, which interact with each other and utilize full information about their environment with the progress of algorithm. For example, honey bees are capable of guaranteeing the survival of a colony without any external guidance. In other words, no one tells honey bees how and where to find food sources; instead, they cooperatively seek the food sources even that is located far away from their nests. In this category, particle swarm optimization (PSO) [6], ant colony optimization (ACO) [7], and artificial bee colony algorithm (ABC) [8] can be regarded as representative algorithms. Some other popular swarm intelligence algorithms are firefly mating algorithm (FMA) [9], shuffled frog leaping algorithm (SFLA) [10], bee collecting pollen algorithm (BCPA) [11], cuckoo search (CS) algorithm [12], dolphin partner optimization (DPO) [13], bat-inspired algorithm (BA) [14], firefly algorithm (FA) [15], and hunting search (HUS) algorithm [16]. Some of the recent swarm intelligence algorithms are fruit fly optimization algorithm (FOA) [17], dragonfly algorithm (DA) [18], artificial algae algorithm (AAA) [19], ant lion optimizer (ALO) [20], shark smell optimization algorithm (DSOA) [21], whale optimization algorithm (WOA) [22], crow search algorithm (CSA) [23], grasshopper optimization algorithm (GOA) [24], mouth brooding fish algorithm (MBFA) [25], spotted hyena optimizer (SHO) [26], butterfly-inspired algorithm (BFA) [27], squirrel search algorithm (SSA) [28], Andean condor algorithm (ACA) [29], and pity beetle algorithm (PBA) [30].

The third category is physical-based algorithms which are based on the basic physical laws such as gravitational force, electromagnetic force, and inertia force. Some of the prevailing algorithms of this category are simulated annealing (SA) [31], gravitational search algorithm (GSA) [32], big-bang big-crunch (BBBC) algorithm [33], charged system search (CSS) [34], black hole (BH) algorithm [35], central force optimization (CFO) [36], small-world optimization algorithm (SWOA) [37], artificial chemical reaction optimization algorithm (ACROA) [38], ray optimization (RO) algorithm [39], galaxy-based search algorithm (GbSA) [40], and curved space optimization (CSO) [41], gravitational search algorithm (GSA) [32], and multiverse optimizer (MVO) [42].

Regardless of the difference among the three categories of algorithms, a common point lies in that besides tuning of common control parameters such as population size and number of generations, the metaheuristic algorithms necessitate tuning of algorithm-specific parameters during the course of optimization. For instance, GA requires tuning of cross-over probability, mutation probability, and selection operator [43]; SA requires tuning of initial temperature and cooling rate [31]; PSO requires tuning of inertia weight and learning factors [6]. The improper tuning of these parameters either increases the computational cost or leads to the local optimal solution.

Recently, a new physical-based metaheuristic algorithm named lightning attachment procedure optimization (LAPO) [44] was proposed, which does not require tuning of any algorithm-specific parameters. Instead, an average value of all solutions was employed to adjust the lightning jump behavior of moving towards or away from a jumping point (or position) in a self-adaptive manner. This is an important reason that LAPO is not easily stuck in the local optimal solution and has a good exploration and exploitation abilities. LAPO has already proved its superiority in solving a number of constrained numerical optimization problems [44].

In this paper, an enhanced lightning attachment procedure optimization, namely, ELAPO is developed to increase the convergence speed during the search process of LAPO while maintaining the key feature of the LAPO as free from algorithm-specific parameters tuning. In ELAPO, a concept of opposition-based learning (OBL) is incorporated for enhancing the searching ability of metaheuristic algorithms. The motivation is that the current estimates and their corresponding opposites are considered simultaneously to find the better solutions, thereby enabling the algorithm to explore a large region of the search space in every generation. This concept was found to be effective in improving the performance of well-known optimization algorithms such as genetic algorithms (GA) [45], differential evolution (DE) [46, 47], particle swarm optimization (PSO) [48, 49], biogeography-based optimization (BBO) [50, 51], harmony search (HS) algorithm [52, 53], gravitational search optimization (GSO) [54, 55], group search algorithm (GSA) [56, 57], and artificial bee colony (ABC) [58]. Meanwhile, a dimensional search strategy is proposed to intensively exploit a local search for each variable of the best solution in each iteration, thus resulting in a higher quality of solution at the end of iteration and strengthening the exploitation of the algorithm. To evaluate the effectiveness of the proposed algorithms, ELAPO is applied to 32 benchmark functions and compared with the basic LAPO and six representative swarm intelligence algorithms (SSA [28], Jaya [59], IBB-BC [60], ODE1 [61], and ALO [20]). The effectiveness of the two strategies is also discussed.

The rest of this paper is organized as follows: Section 2 briefly recapitulates the basic LAPO. Next, the proposed ELAPO is presented in a detailed way in Section 3. Numerical comparisons are illustrated in Section 4. Finally, Section 5 gives the concluding remarks.

## 2. Basic Algorithm

LAPO is a new nature-inspired global optimization, which mimics the lightning attachment procedure including the downward leader movement and the upward leader propagation. The lightning is a sudden electrostatic discharge occurring between electrically charged regions of a cloud, which moves toward or away from the ground in a stepwise movement. After each step, the downward leader stops and then moves to a randomly selected potential point that may have higher value of electrical field. The upward leader starts from sharp points and goes towards the downward leader. The branch fading feature of lightning is taken effect when the charge of a branch is lower than a critical value. In the case where the two leaders join together, a final strike occurs and the charge of the cloud is neutralized.

### 2.1. Parameters and Initialization of Test Points

Main parameters of the LAPO consist of the maximum number of iterations Iter_max_, the number of test points *N*pop, the number of decision variables *n*, and the upper and lower bounds for decision variable *X*_max_ and *X*_min_. These parameters are given at the beginning of the algorithm. Similar to other nature-inspired optimization algorithms, an initial population is required. Each population is regarded as a test point in the feasible search space, which could be an emitting point of the downward or upward leader. The test points are randomly initialized as follows:

(1) $$\begin{array}{c} X_{i,j}=X_{\min}+\text{rand}\left(\;\right)*\left(X_{\max}-X_{\min}\right),\;i=1,\;2,\;\ldots ,\;N\text{pop},\;j=1,\;2,\;\ldots ,\;n, \end{array}$$

where rand( ) is a uniformly distributed random number in the range [0, 1]. The electric field (i.e., fitness value) *f*=(*f*_1_,  *f*_2_,   …,  *f*_*N*pop_) of each test point is calculated based on the objective function:

(2) $$\begin{array}{c} f_{i}=\text{obj}\left(X_{i,1},X_{i,2},\ldots ,X_{i,n}\right),\;\;i=1,\;2,\;\ldots ,\;N\text{pop.} \end{array}$$

### 2.2. Downward Leader Movement toward the Ground

In this phase, all the test points are considered as the downward leader and move down towards the ground. The average value of all test points and its corresponding fitness value are calculated as follows:

(3) $$\begin{array}{c} X_{\text{ave}}=\text{mean}\left(X_{i,j}\right), \end{array}$$

(4) $$\begin{array}{c} f_{\text{ave}}=\text{obj}\left(X_{\text{ave}}\right). \end{array}$$

Given the fact that the lightning has a random behavior, for test point *i*, a random point *k* is selected among the population (*i* ≠ *k*), and the new test point is updated based on the following rules: (i) if the electric field of point *k* is higher than the average electric field, then

(5) $$\begin{array}{c} X_{i,j}^{\text{new}}=X_{i,j}+\text{rand}\left(\;\right)*\left(X_{\text{ave}}-\text{rand}\left(\;\right)*X_{k,j}\right), \end{array}$$

and (ii) if the electric field of point *k* is lower than the average electric field, then

(6) $$\begin{array}{c} X_{i,j}^{\text{new}}=X_{i,j}-\text{rand}\left(\;\right)*\left(X_{\text{ave}}-\text{rand}\left(\;\right)*X_{k,j}\right). \end{array}$$

If the electric field of the new test point is better than the old one, the branch sustains; otherwise, it fades. This feature is mathematically formulated as

(7) $$\begin{array}{c} X_{i,j}=\left\{\begin{array}{cc} X_{i,j}^{\text{new}}, & \text{if }f\left(X_{i,j}^{\text{new}}\right)<f\left(X_{i,j}\right),\\ X_{i,j}, & \text{otherwise.} \end{array}\right. \end{array}$$

### 2.3. Upward Leader Movement

In the upward movement phase, all the test points are considered as the upward leader towards the cloud. The new test points are generated as follows:

(8) $$\begin{array}{c} X_{i,j}^{\text{new}}=X_{i,j}+\text{rand}\left(\;\right)*S*\left(X_{\text{best}}-X_{\text{worst}}\right), \end{array}$$

where *X*_best_ and *X*_worst_ are the best and the worst solutions of the population and *S* is an exponent factor that is a function of the number of iterations Iter and the maximum number of iterations Iter_max_:

(9) $$\begin{array}{c} S=1-\left(\frac{\text{Iter}}{{\text{Iter}}_{\text{max}}}\right)*\exp\left(\frac{\text{Iter}}{{\text{Iter}}_{\text{max}}}\right). \end{array}$$

From a computational point of view, this iteration-dependent exponent factor is important for the balance of exploration and exploitation capabilities of the algorithm. Similar to the downward movement, the branch fading feature also occurs in this phase.

### 2.4. Enhancement of the Performance

In order to enhance the performance of LAPO, in each iteration, the worst test point is replaced by the average test point if the fitness of the former is worse than the latter:

(10) $$\begin{array}{c} X_{\text{worst}}=X_{\text{ave}},\;\;\text{if }f_{\text{ave}}<f\left(X_{\text{worst}}\right). \end{array}$$

### 2.5. Stopping Criterion

The algorithm terminates if the maximum number of iterations is satisfied. Otherwise, the procedures of downward and upward leader movements and of performance enhancement are repeated.

### 2.6. Procedure of the Basic LAPO

The complete computational procedure of the basic LAPO is provided in Algorithm 1.

## 3. The Enhanced Lightning Attachment Procedure Optimization

The enhanced lightning attachment procedure optimization (ELAPO) is presented in this section. Two main strategies exist in the ELAPO. First, a quasi-opposition-based learning strategy is developed and employed randomly to diversify the population. Second, the dimensional search strategy is proposed to improve the quality of the best solution in each iteration. The key ideas behind ELAPO are illustrated as follows.

### 3.1. Quasi-Opposition-Based Learning

In order to prevent the proposed algorithm from being trapped in local optimal solutions, a monitoring condition is introduced and checked in each iteration. Following steps are involved. First, a distance constant between the average test point and the best test point is calculated:

(11) $$\begin{array}{c} D_{\text{c}}=\sqrt{\overset{n}{\underset{j=1}{\sum }}{\left(X_{\text{ave},j}-X_{\text{best},j}\right)}^{2}}. \end{array}$$

Second, the minimum value of the distance constant condition is computed:

(12) $$\begin{array}{c} D_{\text{cmin}}=\frac{15}{10^{\left(\text{Iter}/\left({\text{Iter}}_{\text{max}}\right)\right)}}, \end{array}$$

and the monitoring condition is then checked. If *D*_c_ < *D*_cmin_, the concept of opposition-based learning is employed to further diversify the population and improve the convergence rage of the algorithm. In the strategy, a portion of test points is randomly selected, based on which the corresponding opposite test points are generated and both are considered at the same time. Then, the fitness of the original test points and the quasi-opposite test points are calculated and ranked in a descending order, from which the first *N*pop solutions are selected for proceeding the downward leader movement and the upward leader movement. In order to maintain the stochastic nature of ELAPO, a quasi-opposite solution is randomly generated between the center of the search space CS and the mirror point of the corresponding test point MP:

(13) $$\begin{array}{c} X_{i,j}^{q}=\left\{\begin{array}{c} \begin{array}{cc} \text{CS}+\text{rand}\left(\;\right)*\left(\text{MP}-\text{CS}\right), & \text{if MP}>\text{CS,}\\ \text{MP}+\text{rand}\left(\;\right)*\left(\text{CS}-\text{MP}\right), & \text{ otherwise,} \end{array}\\ i=1,\;2,\;\ldots ,\;Nq, \end{array}\right.\\ \text{CS}=\frac{X_{\max}+X_{\min}}{2},\\ \text{MP}=X_{\max}+X_{\min}-X_{i,j}, \end{array}$$

where *Nq* is the number of randomly chosen test points for the generation of opposite test points, and it is set to be 5 in this paper.

### 3.2. Enhancing Dimensional Search

During the search process of the basic LAPO, all dimensions of each test point are updated simultaneously after each iteration. In other words, different variables in each dimension are dependent. However, this procedure has one obvious drawback: the change in one dimensional variable may cause negative impacts on other dimensional variables, thereby leading to poor convergence performance in each dimension. In order to enhance the dimensional search for each variable, the following four steps are carried out in each iteration: (a) find the best test point, (b) generate one new solution based on the best test point in a way that the value of one variable is revised while the rest of variables are preserved, (c) compare fitness values of the new-generated solution with the old solution, and reserve the better one, and (d) repeat steps (b) and (c) for other dimensional variables. The new-generated solution is produced according the following rule:

(14) $$\begin{array}{c} X_{\text{best},j}^{\text{new}}=X_{\text{best},j}+\text{rand}\left(\;\right)*S*\left(X_{\text{best},j}-X_{\text{worst},j}\right),\;\;j=1,\;2,\;\ldots ,\;n. \end{array}$$

### 3.3. Procedure of ELAPO

The complete computational procedure of the enhanced ELAPO is provided in Algorithm 2.

## 4. Experimental Results and Analysis

In this section, the performance of ELPAO is evaluated by means of 32 different benchmark functions and the results are compared to those of several state-of-the art metaheuristic optimization algorithms. The benchmark functions are listed in Tables 1 [Table tab2]–3, among which F1–F11 are unimodal functions, F13–F25 belong to multimodal functions, and F26–F32 are composite functions provided by IEEE CEC 2014 special section [62]. In these tables, *n* refers to dimension of functions, Range donates the search space, and Fmin is the true optimal value of the test functions. Two kinds of dimension (*n* = 30, and 100) are chosen in order to evaluate the capability of the proposed algorithm for solving different scale test functions.

**Figure 1:**
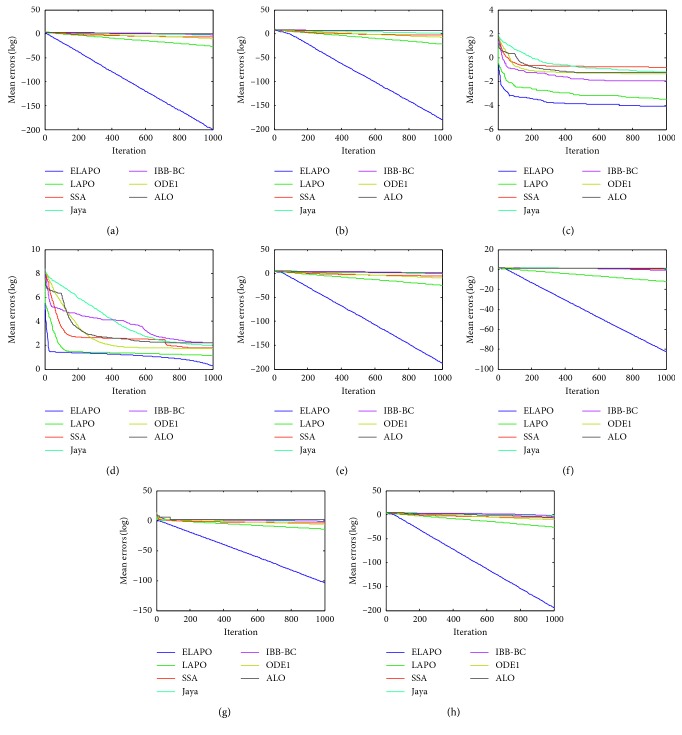
Average convergence curves for the selected unimodal functions (*n*=30). (a) F1. (b) F4. (c) F5. (d) F6. (e) F7. (f) F8. (g) F9. (h) F10.

**Figure 2 fig2:**
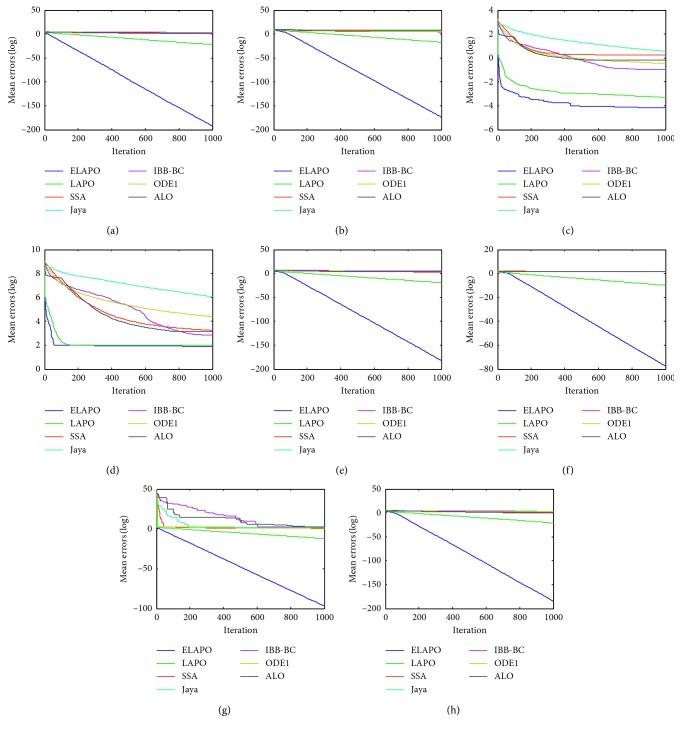
Average convergence curves for the selected unimodal functions (*n*=100). (a) F1. (b) F4. (c) F5. (d) F6. (e) F7. (f) F8. (g) F9. (h) F10.

**Figure 3 fig3:**
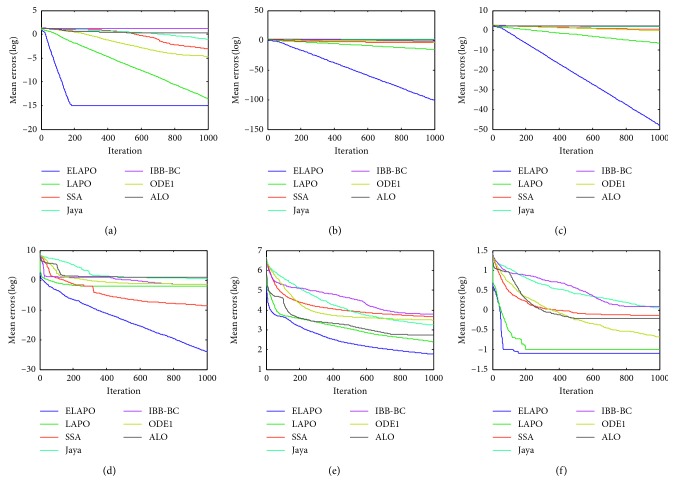
Average convergence curves for the selected multimodal functions (*n*=30). (a) F12. (b) F13. (c) F14. (d) F16. (e) F19. (f) F23.

**Figure 4 fig4:**
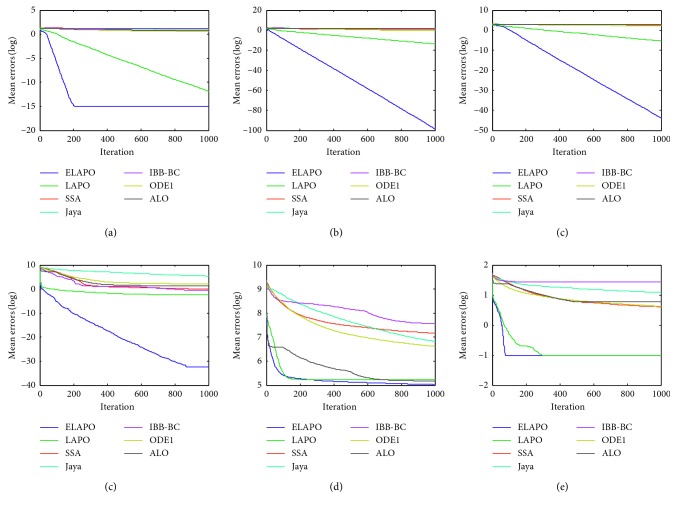
Average convergence curves for the selected multimodal functions (*n*=100). (a) F12. (b) F13. (c) F14. (d) F16. (e) F19. (f) F23.

**Figure 5 fig5:**
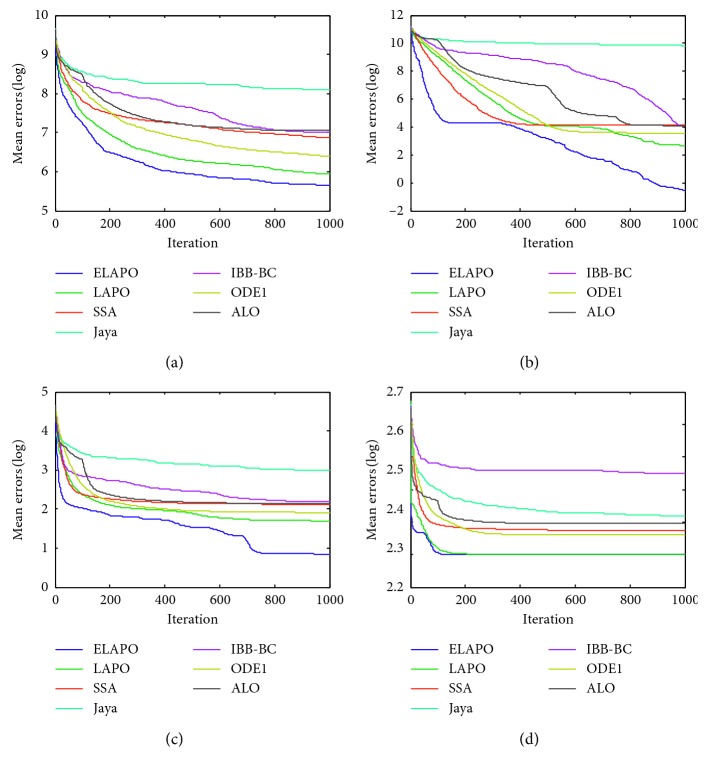
Average convergence curves for the selected CEC 2014 functions (*n*=30). (a) F26. (b) F27. (c) F28. (d) F31.

**Figure 6:**
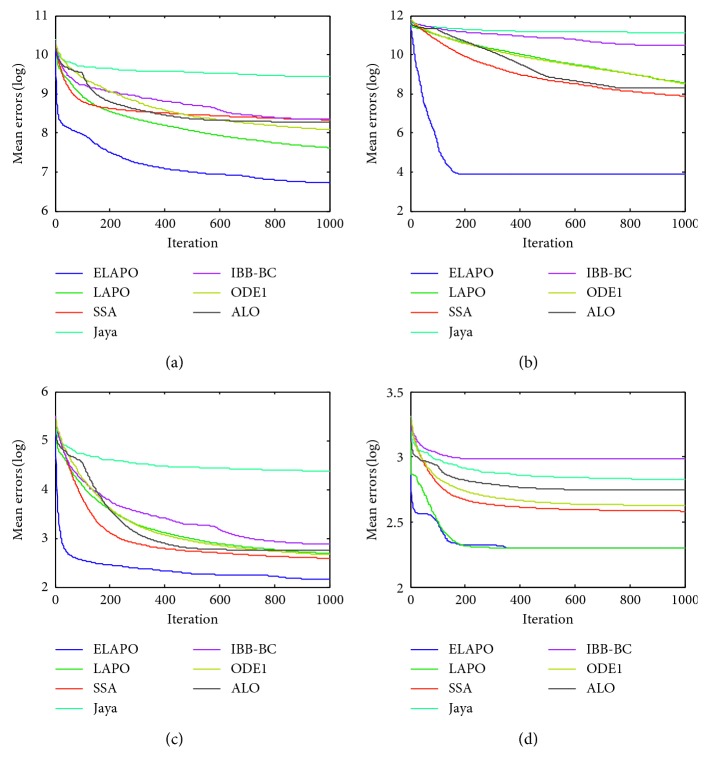
Average convergence curves for the selected CEC 2014 functions (*n*=100). (a)F26. (b) F27. (c) F28. (d) F31.

Six metaheuristic optimization algorithms are utilized in this section as a comparison with the proposed algorithm, including the basic LAPO, squirrel search algorithm (SSA) [28], Jaya [59], improved big bang-big crunch algorithm (IBB-BC) [60], opposition-based differential evolution algorithm (ODE1) [61], and ant lion optimizer (ALO) [20]. The population size and the maximal iteration number are set to be 50 and 1000, respectively. The same set of initial random populations is used to evaluate different algorithms. The error value, defined as *f*(*x*) − *F*_min_, is recorded for the solution *x*, where *f*(*x*) is the optimal fitness value of the function calculated by the algorithms. The widely used parametric settings of all algorithms are listed in Table 4. Each algorithm is applied on the test functions in 10 independent runs. The average and standard deviation of the error values over all independent runs is calculated. Meanwhile, all algorithms are compared in terms of convergence behavior with different curves (Figures 1 [Fig fig2] [Fig fig3] [Fig fig4] [Fig fig5]–6). In addition, the effectiveness of each strategy is tested.

### 4.1. Experimental Test 1: Unimodal Functions

Unimodal benchmark functions have one global optimal solution, and they are commonly used for evaluating the exploitation ability of optimization algorithms. Tables 5 and 6 list the statistical results (the mean error and standard deviation) by different algorithms through 10 independent runs at *n* = 30 and 100, respectively. In these tables, the best values are highlighted in bold. It is obvious from the results that ELAPO has an extremely high level of accuracy and convergence precision for most of unimodal functions in comparison to other six counterpart algorithms. Taken F10 as an example, ELAPO can reach a mean error level of 10*E* − 195 with zero standard deviation at *n*=30, while the accuracy of the rest algorithms is ranked in an order of LAPO (10*E* − 27), ODE1 (10*E* − 11), ALO (10*E* − 7), SSA (10*E* − 6), IBB-BC (10*E* − 4), and Jaya (10*E* − 2). It is also found that ELAPO is able to achieve the true minimal value of F3 and F11, while the rest of algorithms fail to obtain the same level of accuracy except for LAPO on F3. The increase in the number of dimensions seems to not affect the outstanding accuracy of ELAPO in comparison to other algorithms, though the accuracy of all algorithms tends to decrease.

Figures 1 and 2 show the convergence behaviors of some test functions for ELAPO and its competitors at *n* = 30 and 100, respectively. As can be seem from these figures, for most of test functions, ELAPO dramatically outperforms its competitors in terms of both convergence rate and precision. For F5 and F6, the convergence performance of ELAPO is still the best, though LAPO tends to have similar behaviors, and the difference between ELAPO and the rest five algorithms seems to be not very significant. Such excellent performance of ELAPO may be due to the introduction of quasi-opposition-based learning strategy as well as the dimensional search strategy.

### 4.2. Experimental Test 2: Multimodal Functions

Different from unimodal function, multimodal test functions have multiple local optimal solutions and thus are commonly adopted by researchers for testing the exploration ability of an algorithm. Tables 7 and 8 provide the recorded results of statistical analysis over 10 independent runs for *n* = 30 and 100, respectively. From these tables, it is clear that ELAPO can get better level of accuracy for most of test functions compared with other six algorithms. Particularly, ELAPO is able to obtain the exact true values of F17, F18, F21, F22, and F24. It is also interesting to find that ELAPO is still better than LAPO on F20 although both cannot match with SSA at all dimensions involved. Similar to the observation in the unimodal functions, ELAPO seems to be insensitive to the increase of dimensional number.

The convergence performance of all algorithms for several multimodal benchmark functions at *n* = 30 and 100 are presented in Figures 3 and 4, respectively. As can be found in these figures, ELAPO always has the fastest convergence rate and can reach the best (at least comparable) convergence precision in comparison to other six algorithms. For some multimodal functions such as F13, F14, and F16, the convergence performance of LAPO is unsatisfactory, while the global convergence ability of ELAPO is improved greatly. This is mainly contributed by the quasi-opposition-based strategy in which new opposite test points are generated according to a portion of randomly selected test points and both are simultaneously employed for global searching.

### 4.3. Experimental Test 3: CEC 2014 Benchmark Functions

In this experimental study, most intensely investigated benchmark functions used in IEEE CEC 2014 are considered for evaluating both exploration and exploitation capabilities of ELAPO. Seven CEC 2014 functions are considered, which consist of several novel basic problems (e.g., with shifting and rotation) and hybrid and composite test problems. These modern benchmark functions are specially developed with complex features; consequently, all the algorithms can hardly reach the global optimum. However, as per statistical results obtained from different algorithms through 10 independent runs in Tables 9 and 10, ELAPO is able to yield highly competitive results for all CEC 2014 functions under consideration as compared with other six algorithms. For example, the mean error of F27 is as low as a level of 10*E* − 1 for ELAPO, while the corresponding mean errors are dramatically larger for LAPO (10*E* + 2), ODE1 (10*E* + 3), SSA, IBB-BC and ALO (10*E* + 4), and Jaya (10*E* + 9). As the number of dimension increases, all algorithms produce relatively larger mean errors for F26–29 with higher standard deviations, among which ELAPO still ranks No. 1. For the composite functions (F30–32), the increase of dimension seems to not affect the statistical results of all algorithms and ELAPO tends to give slightly better results than other six algorithms.

Figures 5 and 6 show the average convergence curves for four selected CEC 2014 benchmark functions at *n* = 30 and 100, respectively. It is clear that ELAPO has promising convergence behavior compared with other six algorithms, and thus ELAPO proves to be the best among all algorithms on seven CEC 2014 functions. This serves as a further confirmation that ELAPO possesses excellent balance between exploration and exploitation.

### 4.4. Effectiveness of the Two Strategies

In order to verify the effectiveness of the two strategies in the proposed algorithm, this subsection performs the previous three experiments for ELAPO, LAPO with quasi-opposition-based learning only (denoted as ELAPO1), and LAPO with dimensional search strategy only (denoted as ELAPO2), respectively. The statistical results (the minimum, mean and maximum fitness values, and the standard deviation) of different ELAPO variants are recorded in Tables 11 [Table tab12] [Table tab13] [Table tab14] [Table tab15]–16 for various test functions at *n* = 30 and 100. For each function, the overall best results among the three algorithms are highlighted in bold.

For most of the unimodal functions, as shown in Tables 11 and 12, ELAPO outperforms the other two variants in terms of the minimum, mean and maximum fitness values, and the standard deviation. This confirms that for most of the functions, both strategies take effects on enhancing the global search ability and the contribution of the quasi-opposition-based learning strategy is more important. As for F6, it seems that the dimensional search strategy has bigger contributions on the exploitation ability of ELAPO. It is also noted that ELAPO and its two variants have almost the same statistical results because, as per Table 5, the basic LAPO has already converged to desirable accuracy and thus the two strategies seem to have no effects.

Tables 13 and 14 show the minimum, mean and maximum fitness values, and the standard deviation of the multimodal benchmark functions using ELAPO and its two variants. It is clear to find that for most of benchmark functions, both strategies are beneficial to the global search performance and their individual contributions vary for a specific function. For example, the quasi-opposition-based learning strategy has more important effects on F13, F14, and F17; the dimensional search strategy plays a more important role on F15, F16, and F20; both strategies have almost equal contributions on F12 and F23. For F21, F22, and F24, both strategies seem to have no effect. This is because, as per Table 8, the basic LAPO has already obtained the exact optimum. It is also interesting to find that negative effect may be exerted on the global optimization capability. Taking F25 as an example, the best minimum, mean, and maximum fitness values all lied on ELAPO2. In other words, the dimensional search strategy and the quasi-opposition-based learning strategy take positive and negative effects on F25, respectively; the combination of both (ELAPO) can only achieve a middle place of statistical results between ELAPO1 and ELAPO2. This phenomenon is expected and reasonable according to the “no free lunch” (NFL) theorem [59] that stated that no any single algorithm is able to efficiently solve all optimization problems. For some other multimodal functions, the two strategies are sensitive to the number of dimensions. For instance, the contribution of F18 mainly comes from the quasi-opposition-based learning strategy under the condition at *n*=30, while at *n*=100 the main contribution is from the dimensional search strategy. For F19, ELAPO1 seems to have equal contribution as ELAPO2 at *n*=30, while at *n*=100, the former tends to have negative effect on the global search performance.

The statistical results for CEC 2014 benchmark functions are presented in Tables 15 and 16. It can be seen from the tables that both strategies have positive influences on F26, F27, F28, and F29, and the dimensional search strategy tends to take greater effects especially when the number of dimensions is higher. For composite functions, as the complexity of the function increases, the two strategies are still able to make slight contributions, and thus, as per Tables 9 and 10, ELAPO can get competitive results over all other algorithms.

In summary, equipping with any individual strategy only is insufficient to achieve the desired results, but integrating the two strategies results in excellent performance for most of benchmark functions. This superior performance of ELAPO verifies its appropriate taking care of the exploration-exploitation trade-off problem with the introduction of the proposed two strategies.

### 4.5. Statistical Analysis

In order to analyze the performance of any two algorithms, the Wilcoxon signed-rank test and Friedman test [63] are considered for the present work. The results of Wilcoxon's test for ELAPO against other six algorithms are summarized in Tables 17 and 18 for *n* = 30 and 100, respectively. The test is carried out by considering the best solution of each algorithm on each benchmark function with 10 independent runs and a significance level of *α* = 0.05. In Tables 17 and 18, “+” sign indicates that the reference algorithm outperforms the compared one, “−” sign indicates that the reference algorithm is inferior to the compared one, and “=” sign indicates that both algorithms have comparable performances. The results of last row of the tables show that the proposed ELAPO has a larger number of “+” counts in comparison to other algorithms, confirming that ELAPO is better than the other six compared algorithms under 95% level of significance. The results of the Friedman test are presented in Tables 19 and 20 for *n* = 30 and 100, respectively. The last row of the tables depicts the ranks computed through the Friedman test. As can be seen in the table, ELAPO is the best performing algorithm of seven optimization algorithms.

**Figure 7:**
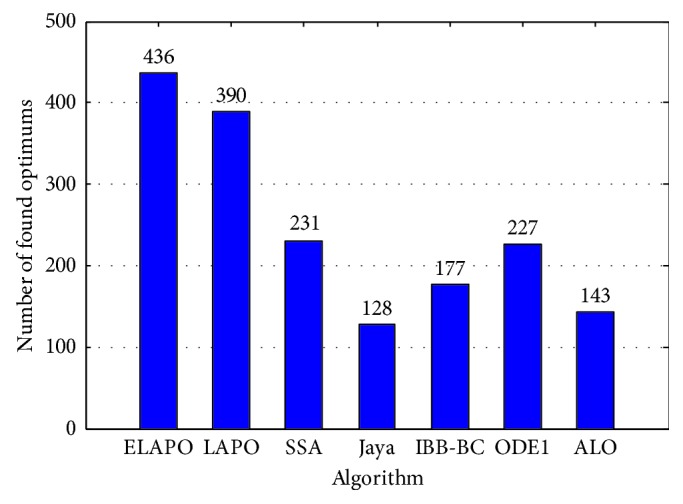
Comparison of algorithms in finding the global optimal solution out of 640 runs.

The quantitative analysis is also carried out for seven algorithms with an index of mean absolute error (MAE) which is an effective performance index for ranking the optimization algorithms and is defined by [64]

(15) $$\begin{array}{c} \text{MAE}=\frac{\sum_{j=1}^{N}\left|m_{j}-k_{j}\right|}{N}, \end{array}$$

where *m*_*j*_ is the mean of optimal values, *k*_*j*_ is the actual global optimal value, and *N* is the number of samples. In the present work, *N* is the number of benchmark functions. The MAE of all algorithms and their ranking for all functions are given in Table 19. It is clear to find that ELAPO ranks No. 1 and provides the minimum MAE in all cases. ELAPO reaches the optimum solution 436 times out of 640 runs (10 runs for each test function for *n* = 30 and 100, respectively) and comes in the first rank as shown in Figure 7. It is concluded that ELAPO provides the best performance in comparison to other six optimization algorithms.

## 5. Conclusions

In this paper, an enhanced lightning attachment procedure optimization called ELAPO is proposed for global optimization problems. The exploration and exploitation abilities of the basic LAPO are appropriately balanced in the search process. The quasi-opposition-based learning strategy is applied to control the convergence speed and to improve both exploration and exploitation abilities of the algorithm. To further enhance the exploitation capability, the dimensional search strategy is employed, which inherits the good information from the best solution in each iteration and thus increases the convergence precision of the proposed algorithm. The efficiency of ELAPO is examined on unimodal, multimodal, and CEC 2014 benchmark functions. The statistic results show that the proposed algorithm has superior performance in terms of accuracy and convergence rates when compared with other six state-of-the-art algorithms including LAPO, SSA, Jaya, IBB-BC, ODE1, and ALO.

## Figures and Tables

**Algorithm 1:**
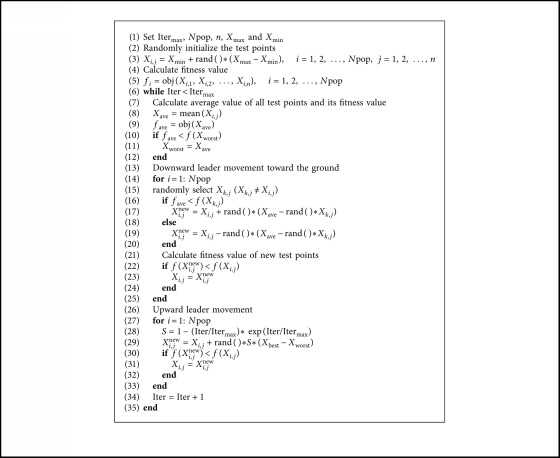
Pseudocode of basic LAPO.

**Algorithm 2:**
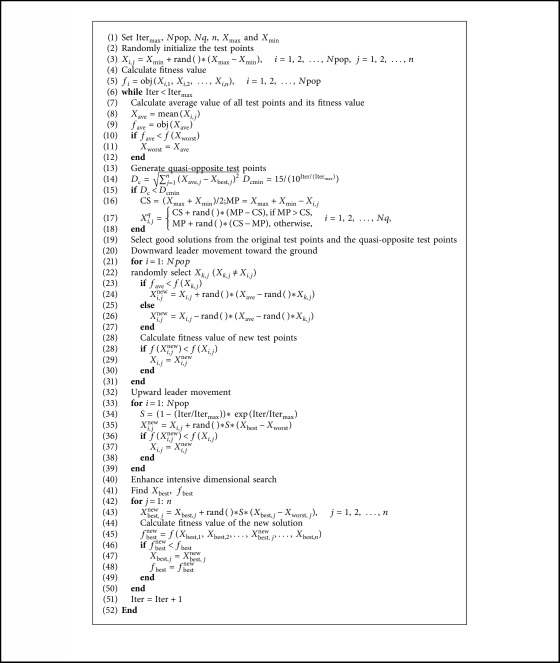
Pseudocode of ELAPO.

**Table 1:** Unimodal benchmark functions.

Function	*n*	Range	*F*_min_
F1(*x*)=∑_*i*=1_^*n*^ *ix*_*i*_^2^	30, 100	[−10, 10]	0
F2(*x*)=∑_*i*=2_^*n*^ *i*(2*x*_*i*_^2^ − *x*_*i*−1_)^2^+(*x*_1_ − 1)^2^	30, 100	[−10, 10]	0
F3(*x*)=−exp(−0.5∑_*i*=1_^*n*^ *x*_*i*_^2^)	30, 100	[−1, 1]	−1
F4(*x*)=∑_*i*=1_^*n*^(10^6^)^((*i* − 1)/(*n* − 1))^ *x*_*i*_^2^	30, 100	[−100, 100]	0
F5(*x*)=∑_*i*=1_^*n*^ *ix*_*i*_^4^+rand( )	30, 100	[−1.28, 1.28]	0
F6(*x*)=∑_*i*=1_^*n*−1^[100(*x*_*i*+1_ − *x*_*i*_^2^)^2^+(*x*_*i*_ − 1)^2^]	30, 100	[−30, 30]	0
F7(*x*)=∑_*i*=1_^*n*^(∑_*j*=1_^*i*^ *x*_*j*_^2^)	30, 100	[−100, 100]	0
F8(*x*)=max{|*x*_*i*_|, 1 ≤ *i* ≤ *n*}	30, 100	[−100, 100]	0
F9(*x*)=∑_*i*=1_^*n*^|*x*_*i*_|+∏_*i*=1_^*n*^|*x*_*i*_|	30, 100	[−10, 10]	0
F10(*x*)=∑_*i*=1_^*n*^ *x*_*i*_^2^	30, 100	[−100, 100]	0
F11(*x*)=∑_*i*=1_^*n*^|*x*_*i*_|^*i*+1^	30, 100	[−1, 1]	0

**Table 2 tab2:** Multimodal benchmark functions.

Function	*n*	Range	*F*_min_
$\text{F}12\left(x\right)=-20\;\exp\left(-0.2\sqrt{\left(1/n\right)\sum_{i=1}^{n}x_{i}^{2}}\right)-\exp\left(1/n\sum_{i=1}^{n}\cos\left(2\pi x_{i}\right)\right)+20+\exp\left(1\right)$	30,100	[−32, 32]	0
F13(*x*)=∑_*i*=1_^*n*^|*x*_*i*_sin(*x*_*i*_)+0.1*x*_*i*_|	30,100	[−10, 10]	0
F14(*x*)=*f*_*s*_(*x*_1_, *x*_2_)+…+*f*_*s*_(*x*_*n*_, *x*_1_), *f*_*s*_(*x*, *y*)=(*x* ^2^+*y* ^2^)^0.25^[sin^2^(50(*x* ^2^+*y* ^2^)^0.1^)+1]	30,100	[−100, 100]	0
$\text{F}15\left(x\right)=f_{s}\left(x_{1},x_{2}\right)+\ldots +f_{s}\left(x_{n},x_{1}\right),f_{s}\left(x,y\right)=0.5\left(\left(\sin^{2}\left(\sqrt{x^{2}+y^{2}}\right)-0.5\right)/{\left(1+0.001\left(x^{2}+y^{2}\right)\right)}^{2}\right)$	30,100	[−100, 100]	0
$\text{F}16\left(x\right)=\pi /n\left\{\begin{array}{c} 10\;\sin^{2}\left(\pi y_{i}\right)+\sum_{i=1}^{n-1}{\left(y_{i}-1\right)}^{2}\left[1+10\;\sin^{2}\left(\pi y_{i+1}\right)\right]+{\left(y_{n}-1\right)}^{2} \end{array}\right\}+\sum_{i=1}^{n}u\left(x_{i},\;10,\;100,\;4\right),\;y_{i}=1+\left(1/4\right)\left(x_{i}+1\right),\;u\left(x_{i},\;a,\;k,\;m\right)=\left\{\begin{array}{cc} k{\left(x_{i}-a\right)}^{m}, & x_{i}>a,\\ 0, & -a\leq x_{i}\leq a,\\ k{\left(-x_{i}-a\right)}^{m}, & x_{i}>a. \end{array}\right.$	30,100	[−50, 50]	0
$\text{F}17\left(x\right)=\left(1/4000\right)\sum_{i=1}^{n}x_{i}^{2}-\prod_{i=1}^{n}\cos\left(x_{i}/\sqrt{i}\right)+1$	30,100	[−100, 100]	0
$\text{F}18\left(x\right)=-\sum_{i=1}^{n-1}\left(\exp\left(-\left(x_{i}^{2}+x_{i+1}^{2}+0.5x_{i}x_{i+1}\right)/8\right)*\;\cos\left(4\sqrt{x_{i}^{2}+x_{i+1}^{2}+0.5x_{i}x_{i+1}}\right)\right)$	30,100	[−5, 5]	1 − *n*
F19(*x*)=∑_*i*=1_^*n*^(*x*_*i*_ − 1)^2^ − ∑_*i*=2_^*n*^ *x*_*i*_ *x*_*i*−1_	30,100	[−*n* ^2^, *n* ^2^]	(*n*(*n*+4)(*n* − 1))/−6
$\text{F}20\left(x\right)\;=\sum_{i=2}^{n-1}\left(0.5+\left(\sin^{2}\left(\sqrt{100x_{i}^{2}+x_{i+1}^{2}}\right)-0.5\right)\right)/\left({\left(1+0.001\left(x_{i}^{2}-2x_{i}x_{i-1}+x_{i-1}^{2}\right)\right)}^{2}\right)$	30,100	[−100, 100]	0
F21(*x*)=∑_*i*=1_^*n*^[*x*_*i*_^2^ − 10 cos(2*πx*_*i*_)+10]	30,100	[−5.12, 5.12]	0
$\text{F}22\left(x\right)=\sum_{i=1}^{n}\left[y_{i}^{2}-10\;\cos\left(2\pi y_{i}\right)+10\right],\;y_{i}=\left\{\begin{array}{cc} x_{i}, & \left|x_{i}\right|<0.5,\\ \text{round}\left(2x_{i}\right)/2, & \left|x_{i}\right|<0.5. \end{array}\right.$	30,100	[−5.12, 5.12]	0
$\text{F}23\left(x\right)=1-\cos\left(2\pi \sqrt{\sum_{i=1}^{n}x_{i}^{2}}\right)+0.1\sqrt{\sum_{i=1}^{n}x_{i}^{2}}$	30,100	[−100, 100]	0
F24(*x*)=∑_*i*=1_^*n*^{∑_*k*=0_^*k*_max_^[*a* ^*k*^cos(2*πb* ^*k*^(*x*_*i*_+0.5))]} − *n*∑_*k*=0_^*k*_max_^[*a* ^*k*^cos(2*πb* ^*k*^0.5)]	30,100	[−0.5, 0.5]	0
F25(*x*)=∑_*k*=1_^*n*^∑_*j*=1_^*n*^((*y*_*jk*_^2^/4000) − cos(*y*_*jk*_)+1), *y*_*jk*_=100(*x*_*k*_ − *x*_*j*_^2^)^2^+(1 − *x*_*j*_^2^)^2^	30,100	[−100, 100]	0

**Table 3:** CEC 2014 benchmark functions.

Function	*n*	Range	*F*_min_
F26 (CEC1: rotated high-conditioned elliptic function)	30, 100	[−100, 100]	100
F27 (CEC2: rotated bent cigar function)	30, 100	[−100, 100]	200
F28 (CEC4: shifted and rotated Rosenbrock's function)	30, 100	[−100, 100]	400
F29 (CEC17: hybrid function 1)	30, 100	[−100, 100]	1700
F30 (CEC23: composition function 1)	30, 100	[−100, 100]	2300
F31 (CEC24: composition function 2)	30, 100	[−100, 100]	2400
F32 (CEC25: composition function 3)	30, 100	[−100, 100]	2500

**Table 4:** Parameter setting for the involved algorithms.

Algorithm	Parameter
ELAPO	—
LAPO	—
SSA	*G*_c_=1.9, *sf* = 18, *P*_dp_=0.01, *N*_*fs*_ = 4
Jaya	—
IBB-BC	*γ* = 0.2, *α* = 3
ALO	—
ODE1	*F* = 0.5, Cr = 0.9, JR = 0.3

**Table 5:** Statistical results obtained by different algorithms through 10 independent runs for unimodal benchmark functions at *n*=30.

Function		ELAPO	LAPO	SSA	Jaya	IBB-BC	ODE1	ALO
F1	Mean	**7.5637E** − **200**	5.6012*E* − 27	1.1428*E* − 07	2.2752*E* − 03	1.3687*E* − 02	1.0558*E* − 10	4.4514*E* − 01
	Std	**0.0000E** + **00**	8.4361*E* − 27	4.8364*E* − 08	8.0246*E* − 04	1.5007*E* − 02	3.2551*E* − 10	7.8851*E* − 01

F2	Mean	**6.6667E** − **01**	**6.6667E** − **01**	9.4666*E* − 01	1.5883*E* + 00	1.9627*E* + 00	8.2956*E* − 01	1.9322*E* + 00
	Std	**1.1703E** − **16**	**2.6825E** − **15**	9.0549*E* − 01	1.4893*E* + 00	3.0291*E* + 00	3.4162*E* − 01	1.6808*E* + 00

F3	Mean	**0.0000E** + **00**	**0.0000E** + **00**	4.8854*E* − 11	1.0043*E* − 06	9.5870*E* − 01	0.0000*E* + 00	1.1247*E* − 11
	Std	**0.0000E** + **00**	**0.0000E** + **00**	2.4693*E* − 11	3.2914*E* − 07	1.6557*E* − 02	6.3238*E* − 16	5.3013*E* − 12

F4	Mean	**1.8129E** − **180**	3.6621*E* − 22	1.4830*E* − 03	1.5201*E* + 01	3.1383*E* + 06	4.6192*E* − 08	2.4627*E* + 06
	Std	**0.0000E** + **00**	9.1437*E* − 22	1.3430*E* − 03	5.5752*E* + 00	1.5412*E* + 06	9.0067*E* − 08	1.2642*E* + 06

F5	Mean	**8.6010E** − **05**	3.3511*E* − 04	1.4931*E* − 01	6.6240*E* − 02	1.1173*E* − 02	4.5462*E* − 02	5.6409*E* − 02
	Std	**8.9264E** − **05**	2.3261*E* − 04	4.1331*E* − 02	8.9128*E* − 03	1.7186*E* − 03	1.1837*E* − 02	2.0886*E* − 02

F6	Mean	**1.8767E** + **00**	1.3696*E* + 01	6.0642*E* + 01	9.2536*E* + 01	1.6858*E* + 02	5.2937*E* + 01	1.6217*E* + 02
	Std	**8.1532E** − **01**	9.9927*E* − 01	3.5585*E* + 01	4.5618*E* + 01	2.2938*E* + 02	2.9763*E* + 01	1.6493*E* + 02

F7	Mean	**2.5280E** − **188**	1.3524*E* − 25	9.7179*E* − 06	2.7516*E* − 01	7.1328*E* − 01	5.3976*E* − 10	1.7832*E* + 01
	Std	**0.0000E** + **00**	1.3883*E* − 25	5.6762*E* − 06	1.1147*E* − 01	9.4554*E* − 01	1.2392*E* − 09	2.0860*E* + 01

F8	Mean	**5.2137E** − **83**	8.3427*E* − 13	2.6260*E* + 00	6.9426*E* + 00	1.6891*E* − 01	7.1509*E* + 00	8.1078*E* + 00
	Std	**8.6122E** − **83**	5.5630*E* − 13	6.1185*E* − 01	3.4493*E* + 00	1.3435*E* − 01	3.2087*E* + 00	2.2939*E* + 00

F9	Mean	**3.6582E** − **104**	6.5210*E* − 15	1.9378*E* − 04	7.0569*E* − 02	5.0370*E* − 02	7.7739*E* − 07	3.4909*E* + 01
	Std	**5.3056E** − **104**	1.4104*E* − 14	5.6284*E* − 05	2.2004*E* − 02	2.9201*E* − 02	3.0011*E* − 07	4.8480*E* + 01

F10	Mean	**8.1828E** − **195**	5.8616*E* − 27	1.0567*E* − 06	1.9651*E* − 02	3.5197*E* − 04	1.5345*E* − 11	6.6424*E* − 07
	Std	**0.0000E** + **00**	8.6367*E* − 27	8.7349*E* − 07	6.2693*E* − 03	1.4587*E* − 04	1.5332*E* − 11	5.6633*E* − 07

F11	Mean	**0.0000E** + **00**	1.4564*E* − 103	1.3567*E* − 41	3.4416*E* − 13	6.3934*E* − 08	9.7599*E* − 13	1.3755*E* − 07
	Std	**0.0000E** + **00**	3.7807*E* − 103	3.3385*E* − 41	7.5948*E* − 13	6.9210*E* − 08	2.3650*E* − 12	9.0853*E* − 08

**Table 6:** Statistical results obtained by different algorithms through 10 independent runs for unimodal benchmark functions at *n*=100.

Function		ELAPO	LAPO	SSA	Jaya	IBB-BC	ODE1	ALO
F1	Mean	**5.2007E** − **193**	1.3425*E* − 22	1.6628*E* + 01	6.9384*E* + 02	1.8003*E* + 03	1.8499*E* + 01	1.1049*E* + 02
	Std	**0.0000E** + **00**	2.7548*E* − 22	3.1328*E* + 00	1.5143*E* + 02	1.1134*E* + 03	1.2793*E* + 01	3.4927*E* + 01

F2	Mean	**6.6667E** − **01**	**6.6667E** − **01**	9.3976*E* + 01	1.7780*E* + 04	5.8507*E* + 01	2.4492*E* + 02	6.5552*E* + 01
	Std	**1.1703E** − **16**	**3.1465E** − **07**	1.8308*E* + 01	9.2943*E* + 03	5.5585*E* + 01	1.0120*E* + 02	1.6325*E* + 01

F3	Mean	**0.0000E** + **00**	**0.0000E** + **00**	2.1775*E* − 03	8.7150*E* − 02	1.0000*E* + 00	3.7746*E* − 03	1.7589*E* − 06
	Std	**0.0000E** + **00**	**0.0000E** + **00**	4.0246*E* − 04	1.9928*E* − 02	1.4447*E* − 06	4.3774*E* − 03	7.2246*E* − 07

F4	Mean	**3.5068E** − **174**	7.6209*E* − 18	5.7986*E* + 04	3.3097*E* + 06	6.4465*E* + 07	1.1275*E* + 05	3.7806*E* + 07
	Std	**0.0000E** + **00**	1.4480*E* − 17	1.0700*E* + 04	1.8257*E* + 06	1.8345*E* + 07	1.3526*E* + 05	1.0211*E* + 07

F5	Mean	**6.9030E** − **05**	5.0481*E* − 04	1.7809*E* + 00	3.7596*E* + 00	1.1013*E* − 01	3.4775*E* − 01	6.9402*E* − 01
	Std	**1.2101E** − **04**	3.4086*E* − 04	2.8494*E* − 01	1.2333*E* + 00	3.0997*E* − 02	1.6561*E* − 01	8.1848*E* − 02

F6	Mean	**7.8513E** + **01**	9.4177*E* + 01	1.7444*E* + 03	1.1493*E* + 06	7.2389*E* + 02	2.3396*E* + 04	1.4132*E* + 03
	Std	**1.6552E** + **00**	8.9340*E* − 01	5.2182*E* + 02	4.4284*E* + 05	4.8630*E* + 02	2.7369*E* + 04	1.2610*E* + 03

F7	Mean	**1.1968E** − **182**	6.3973*E* − 20	1.8395*E* + 03	6.3193*E* + 04	1.3746*E* + 05	2.9564*E* + 03	1.2062*E* + 04
	Std	**0.0000E** + **00**	1.2555*E* − 19	3.3413*E* + 02	1.2607*E* + 04	7.9074*E* + 04	2.1890*E* + 03	3.5187*E* + 03

F8	Mean	**4.0687E** − **78**	1.5876*E* − 10	5.6812*E* + 01	5.0409*E* + 01	5.3766*E* + 01	2.4541*E* + 01	2.7036*E* + 01
	Std	**3.8556E** − **78**	8.3377*E* − 11	3.3342*E* + 00	4.5067*E* + 00	4.7994*E* + 00	4.2786*E* + 00	3.7373*E* + 00

F9	Mean	**1.7153E** − **97**	6.0151*E* − 13	3.7898*E* + 00	4.0015*E* + 01	2.2845*E* + 02	2.5136*E* + 00	2.5022*E* + 02
	Std	**2.6804E** − **97**	7.2192*E* − 13	3.3142*E* − 01	9.5389*E* + 00	2.4337*E* + 01	1.1042*E* + 00	1.8829*E* + 02

F10	Mean	**2.1712E** − **185**	1.0305*E* − 21	4.5309*E* + 01	2.0729*E* + 03	1.5794*E* + 03	1.5935*E* + 02	7.2968*E* − 01
	Std	**0.0000E** + **00**	1.3847*E* − 21	8.5410*E* + 00	5.9716*E* + 02	1.4768*E* + 03	2.6888*E* + 02	3.5403*E* − 01

F11	Mean	**0.0000E** + **00**	6.1091*E* − 103	4.7120*E* − 29	2.4029*E* − 07	8.6095*E* − 08	1.6291*E* − 10	1.5573*E* − 07
	Std	**0.0000E** + **00**	1.6435*E* − 102	1.1490*E* − 28	3.3712*E* − 07	5.7812*E* − 08	5.1012*E* − 10	1.0685*E* − 07

**Table 7:** Statistical results obtained by different algorithms through 10 independent runs for multimodal benchmark functions at *n*=30.

Fun		ELAPO	LAPO	SSA	Jaya	IBB-BC	ODE1	ALO
F12	Mean	**8.8818E** − **16**	2.8955*E* − 14	9.2571*E* − 04	9.1487*E* − 02	1.7601*E* + 01	2.5302*E* − 05	1.6649*E* + 00
	Std	**0.0000E** + **00**	2.4640*E* − 14	9.2067*E* − 04	7.7490*E* − 02	6.9501*E* − 01	7.7013*E* − 05	7.7554*E* − 01

F13	Mean	**6.1910E** − **102**	2.2608*E* − 16	1.1359*E* − 04	1.8262*E* + 01	5.4072*E* − 02	5.6933*E* − 04	5.2074*E* + 00
	Std	**1.7386E** − **101**	1.6391*E* − 16	5.9272*E* − 05	8.6301*E* + 00	4.1391*E* − 02	1.2644*E* − 03	3.1551*E* + 00

F14	Mean	**1.4029E** − **48**	4.0494*E* − 07	3.4234*E* + 00	5.1721*E* + 01	1.1805*E* + 02	6.8554*E* − 01	1.3231*E* + 02
	Std	**1.7605E** − **48**	2.6617*E* − 07	9.0812*E* − 01	9.8384*E* + 00	1.7959*E* + 01	1.7645*E* − 01	2.0228*E* + 01

F15	Mean	1.4563*E* + 00	9.4103*E* + 00	**9.0346E** − **01**	1.2447*E* + 01	1.2934*E* + 01	1.2364*E* + 01	1.2030*E* + 01
	Std	4.3965*E* − 01	5.3815*E* − 01	**3.2252E** − **01**	2.9666*E* − 01	5.3194*E* − 01	2.5704*E* − 01	7.5729*E* − 01

F16	Mean	**8.4188E** − **25**	1.0367*E* − 02	2.2266*E* − 09	4.1310*E* + 00	4.1888*E* − 02	4.2953*E* − 02	9.0097*E* + 00
	Std	**1.5385E** − **24**	3.2783*E* − 02	1.5417*E* − 09	1.6123*E* + 00	5.4071*E* − 02	5.2502*E* − 02	3.2448*E* + 00

F17	Mean	**0.0000E** + **00**	7.3960*E* − 04	2.2384*E* − 02	2.9603*E* − 01	5.1123*E* − 02	5.4206*E* − 03	1.0343*E* − 02
	Std	**0.0000E** + **00**	2.3388*E* − 03	1.3974*E* − 02	2.2107*E* − 01	6.7979*E* − 02	6.1229*E* − 03	1.0090*E* − 02

F18	Mean	**0.0000E** + **00**	1.6903*E* + 01	5.7452*E* + 00	2.1310*E* + 01	2.5789*E* + 01	2.0659*E* + 01	1.8115*E* + 01
	Std	**0.0000E** + **00**	6.2598*E* + 00	1.6003*E* + 00	8.2816*E* − 01	4.4569*E* − 01	8.8290*E* − 01	2.5728*E* + 00

F19	Mean	**5.8977E** + **01**	2.5151*E* + 02	4.5305*E* + 03	1.7584*E* + 03	6.3780*E* + 03	3.0747*E* + 03	5.5289*E* + 02
	Std	**2.4102E** + **01**	1.0785*E* + 02	3.6141*E* + 03	2.6629*E* + 03	6.1551*E* + 03	1.1640*E* + 03	1.7077*E* + 02

F20	Mean	2.0743*E* − 03	1.5861*E* − 02	**3.1866E** − **04**	7.3750*E* − 02	3.3350*E* − 03	3.9377*E* − 03	8.3297*E* − 03
	Std	2.6236*E* − 03	1.5529*E* − 02	**3.6113E** − **04**	2.9955*E* − 02	1.3663*E* − 03	2.3799*E* − 03	4.6551*E* − 03

F21	Mean	**0.0000E** + **00**	**0.0000E** + **00**	8.1814*E* − 06	2.2150*E* + 02	5.3244*E* + 01	1.5228*E* + 02	7.8900*E* + 01
	Std	**0.0000E** + **00**	**0.0000E** + **00**	1.2639*E* − 05	2.0149*E* + 01	1.7451*E* + 01	2.5607*E* + 01	2.5535*E* + 01

F22	Mean	**0.0000E** + **00**	1.2264*E* + 01	1.6897*E* − 01	2.0120*E* + 02	4.6472*E* + 01	1.1726*E* + 02	8.5910*E* + 01
	Std	**0.0000E** + **00**	2.5856*E* + 01	2.7200*E* − 01	1.4093*E* + 01	1.2995*E* + 01	4.0305*E* + 01	5.0467*E* + 01

F23	Mean	**7.9899E** − **02**	9.9873*E* − 02	7.1987*E* − 01	1.1289*E* + 00	1.2306*E* + 00	2.1167*E* − 01	6.0987*E* − 01
	Std	**4.2110E** − **02**	9.2544*E* − 09	6.3246*E* − 02	7.3969*E* − 02	2.7575*E* − 01	3.1132*E* − 02	7.3786*E* − 02

F24	Mean	**0.0000E** + **00**	**0.0000E** + **00**	2.0537*E* − 02	1.6488*E* + 00	1.4966*E* + 01	1.8835*E* − 02	1.7219*E* + 01
	Std	**0.0000E** + **00**	**0.0000E** + **00**	8.4213*E* − 03	7.2301*E* − 01	4.3592*E* + 00	3.8947*E* − 02	3.2418*E* + 00

F25	Mean	**3.4556E** + **02**	6.2050*E* + 02	3.6018*E* + 02	8.1492*E* + 02	8.1624*E* + 02	7.6337*E* + 02	7.3879*E* + 02
	Std	**3.3722E** + **01**	1.4539*E* + 02	1.1172*E* + 02	1.9261*E* + 01	2.0303*E* + 01	5.3897*E* + 01	3.2218*E* + 01

**Table 8:** Statistical results obtained by different algorithms through 10 independent runs for multimodal benchmark functions at *n*=100.

Fun		ELAPO	LAPO	SSA	Jaya	IBB-BC	ODE1	ALO
F12	Mean	**8.8818E** − **16**	1.2518*E* − 12	1.6404*E* + 01	7.5892*E* + 00	1.8804*E* + 01	3.7259*E* + 00	6.8021*E* + 00
	Std	**0.0000E** + **00**	1.9773*E* − 12	7.3335*E* + 00	6.3212*E* – 01	2.1140*E* − 01	9.9069*E* − 01	1.3361*E* + 00

F13	Mean	**3.6083E** − **99**	2.2834*E* − 14	3.9731*E* + 00	5.3830*E* + 01	5.3426*E* + 01	4.6040*E* − 01	3.6655*E* + 01
	Std	**5.4747E** − **99**	1.5184*E* − 14	1.0066*E* + 00	8.5028*E* + 00	8.0583*E* + 00	3.3718*E* − 01	1.1067*E* + 01

F14	Mean	**1.7929E** − **44**	4.5968*E* − 06	4.1324*E* + 02	6.3538*E* + 02	7.7868*E* + 02	1.4752*E* + 02	6.0052*E* + 02
	Std	**2.8722E** − **44**	2.6509*E* − 06	5.0767*E* + 01	4.8142*E* + 01	3.4226*E* + 01	2.1587*E* + 01	5.0591*E* + 01

F15	Mean	**7.2183E** + **00**	4.0465*E* + 01	2.7036*E* + 01	4.6481*E* + 01	4.4956*E* + 01	4.5921*E* + 01	4.1953*E* + 01
	Std	**8.8821E** + **00**	9.3027*E* − 01	1.9176*E* + 00	3.0431*E* – 01	8.6263*E* − 01	1.0532*E* + 00	2.1483*E* + 00

F16	Mean	**4.7116E** − **33**	3.3016*E* − 03	9.3548*E* − 01	2.7447*E* + 05	3.2179*E* − 01	1.5236*E* + 02	2.2466*E* + 01
	Std	**1.9082E** − **48**	9.8103*E* − 03	3.5109*E* − 01	4.6952*E* + 05	2.2593*E* − 01	2.5273*E* + 02	7.5516*E* + 00

F17	Mean	**0.0000E** + **00**	**0.0000E** + **00**	4.9065*E* − 01	1.4511*E* + 00	2.2854*E* + 00	3.9525*E* − 01	1.7248*E* − 01
	Std	**0.0000E** + **00**	**0.0000E** + **00**	6.1173*E* − 02	9.6738*E* – 02	3.8727*E* − 01	1.6189*E* − 01	3.8321*E* − 02

F18	Mean	**2.4937E** + **01**	8.3464*E* + 01	2.9867*E* + 01	8.6792*E* + 01	9.2907*E* + 01	8.6137*E* + 01	6.7602*E* + 01
	Std	**3.2165E** + **01**	3.3841*E* + 00	2.0862*E* + 00	1.0848*E* + 00	1.0617*E* + 00	9.8553*E* − 01	6.6475*E* + 00

F19	Mean	**1.0585E** + **05**	1.6900*E* + 05	1.4604*E* + 07	6.4959*E* + 06	3.7560*E* + 07	4.1557*E* + 06	1.5224*E* + 05
	Std	**4.1853E** + **04**	2.9643*E* + 02	4.0214*E* + 06	1.5787*E* + 06	1.4721*E* + 07	1.1991*E* + 06	1.1620*E* + 04

F20	Mean	7.3630*E* − 02	1.5070*E* + 00	**4.4115E** − **02**	2.8379*E* + 00	2.9689*E* − 01	1.7898*E* − 01	3.3727*E* − 01
	Std	1.5569*E* − 01	3.5022*E* − 01	**1.4144E** − **02**	4.1322*E* – 01	1.3280*E* − 01	5.3857*E* − 02	9.9795*E* − 02

F21	Mean	**0.0000E** + **00**	**0.0000E** + **00**	1.3590*E* + 02	9.3314*E* + 02	4.9346*E* + 02	7.0721*E* + 02	2.4664*E* + 02
	Std	**0.0000E** + **00**	**0.0000E** + **00**	1.1534*E* + 01	5.9504*E* + 01	4.5275*E* + 01	8.4180*E* + 01	5.1974*E* + 01

F22	Mean	**0.0000E** + **00**	**0.0000E** + **00**	9.0892*E* + 01	9.5631*E* + 02	5.4737*E* + 02	7.5311*E* + 02	4.1579*E* + 02
	Std	**0.0000E** + **00**	**0.0000E** + **00**	1.3962*E* + 01	6.2407*E* + 01	5.9740*E* + 01	4.7769*E* + 01	1.0978*E* + 02

F23	Mean	**9.9873E** − **02**	**9.9873E** − **02**	4.0825*E* + 00	1.2141*E* + 01	2.7545*E* + 01	4.1911*E* + 00	5.8799*E* + 00
	Std	**7.7820E** − **17**	**9.5023E** − **09**	4.1814*E* − 01	8.9201*E* – 01	1.7224*E* + 00	5.9174*E* − 01	8.4301*E* − 01

F24	Mean	**0.0000E** + **00**	**0.0000E** + **00**	1.5556*E* + 01	5.0470*E* + 01	1.2573*E* + 02	1.0650*E* + 01	8.9877*E* + 01
	Std	**0.0000E** + **00**	**0.0000E** + **00**	2.0040*E* + 00	3.8831*E* + 00	6.1093*E* + 00	1.7570*E* + 00	6.6157*E* + 00

F25	Mean	**3.6192E** + **03**	4.5995*E* + 03	1.0766*E* + 04	3.5395*E* + 06	1.0381*E* + 04	6.5557*E* + 04	1.2502*E* + 04
	Std	**8.1059E** + **02**	9.5869*E* − 13	2.6120*E* + 02	1.8511*E* + 06	2.2635*E* + 02	6.1928*E* + 04	6.4827*E* + 02

**Table 9:** Statistical results obtained by different algorithms through 10 independent runs for CEC 2014 benchmark functions at *n*=30.

Fun		ELAPO	LAPO	SSA	Jaya	IBB-BC	ODE1	ALO
F26	Mean	**4.4625E** + **05**	8.6316*E* + 05	7.4184*E* + 06	1.2764*E* + 08	1.0313*E* + 07	2.4740*E* + 06	1.1687*E* + 07
	Std	**4.1890E** + **05**	4.7927*E* + 05	4.2619*E* + 06	4.7324*E* + 07	7.9033*E* + 06	2.0985*E* + 06	4.7916*E* + 06

F27	Mean	**3.0019E** − **01**	4.8777*E* + 02	1.2834*E* + 04	6.8890*E* + 09	1.2215*E* + 04	3.6652*E* + 03	1.2669*E* + 04
	Std	**5.2289E** − **01**	4.4184*E* + 02	1.1687*E* + 04	8.9349*E* + 08	6.3267*E* + 03	5.1977*E* + 03	7.5825*E* + 03

F28	Mean	**7.0443E** + **00**	4.9654*E* + 01	1.2784*E* + 02	9.7486*E* + 02	1.5431*E* + 02	7.9861*E* + 01	1.3876*E* + 02
	Std	**2.1148E** + **01**	3.3024*E* + 01	3.9603*E* + 01	1.8138*E* + 02	1.0217*E* + 01	1.9594 *E* + 01	4.1850*E* + 01

F29	Mean	1.6076*E* + 05	2.9803*E* + 05	1.2743*E* + 06	6.8526*E* + 06	6.1188*E* + 05	**2.4020E** + **04**	1.1616*E* + 06
	Std	1.0464*E* + 05	1.4700*E* + 05	6.7634*E* + 05	3.2198*E* + 06	4.6454*E* + 05	**2.4324E** + **04**	7.6552*E* + 05

F30	Mean	**3.1524E** + **02**	**3.1524E** + **02**	3.1526*E* + 02	3.6242*E* + 02	4.0373*E* + 02	3.1525*E* + 02	3.2299*E* + 02
	Std	**4.7935E** − **13**	**9.3896E** − **12**	1.5792*E* − 02	1.1067*E* + 01	3.7105*E* + 01	3.7343*E* − 03	3.8656*E* + 00

F31	Mean	**2.0000E** + **02**	2.0001*E* + 02	2.3645*E* + 02	2.6188*E* + 02	3.5442*E* + 02	2.2897*E* + 02	2.4859*E* + 02
	Std	**3.5666E** − **04**	1.7300*E* − 03	8.9457*E* + 00	8.8881*E* + 00	1.5967*E* + 01	5.5403*E* + 00	5.1698*E* + 00

F32	Mean	**2.0000E** + **02**	**2.0000E** + **02**	2.0932*E* + 02	2.2711*E* + 02	2.4501*E* + 02	2.0312*E* + 02	2.2389*E* + 02
	Std	**0.0000E** + **00**	**2.1437E** − **13**	5.7620*E* + 00	3.4992*E* + 00	1.1029*E* + 01	3.2954*E* − 01	4.8761*E* + 00

**Table 10:** Statistical results obtained by different algorithms through 10 independent runs for CEC 2014 benchmark functions at *n*=100.

Fun		ELAPO	LAPO	SSA	Jaya	IBB-BC	ODE1	ALO
F26	Mean	**5.2160E** + **06**	4.1519*E* + 07	2.1696*E* + 08	2.7847*E* + 09	2.2334*E* + 08	1.1944*E* + 08	1.9248*E* + 08
	Std	**3.1061E** + **06**	1.0226*E* + 07	6.2278*E* + 07	5.3126*E* + 08	4.9251*E* + 07	4.6916*E* + 07	5.2322*E* + 07

F27	Mean	**7.6528E** + **03**	3.6048*E* + 08	7.7122*E* + 07	1.3095*E* + 11	2.9545*E* + 10	3.1169*E* + 08	1.9225*E* + 08
	Std	**4.6668E** + **03**	4.0022*E* + 08	1.5673*E* + 07	1.4234*E* + 10	1.6259*E* + 10	2.7877*E* + 08	1.2626*E* + 08

F28	Mean	**1.4633E** + **02**	4.8679*E* + 02	3.8588*E* + 02	2.3145*E* + 04	7.7160*E* + 02	4.5681*E* + 02	5.5644*E* + 02
	Std	**4.9530E** + **01**	8.2735*E* + 01	4.7462*E* + 01	4.8763*E* + 03	1.1464*E* + 02	6.2699*E* + 01	5.1636*E* + 01

F29	Mean	**2.0346E** + **06**	3.8720*E* + 06	2.3420*E* + 07	2.7397*E* + 08	9.2325*E* + 06	9.7017*E* + 06	8.6593*E* + 06
	Std	**1.2767E** + **06**	2.1761*E* + 06	1.3998*E* + 07	3.4244*E* + 07	4.6827*E* + 06	2.9845*E* + 06	3.3179*E* + 06

F30	Mean	**3.4508E** + **02**	3.5079*E* + 02	3.5220*E* + 02	1.1047*E* + 03	9.8448*E* + 02	3.5205*E* + 02	4.8240*E* + 02
	Std	**6.2281E** + **01**	1.2145*E* + 00	2.1181*E* + 00	1.5451*E* + 02	1.4673*E* + 02	3.0127*E* + 00	2.5739*E* + 01

F31	Mean	**2.0000E** + **02**	2.0002*E* + 02	3.8192*E* + 02	6.6879*E* + 02	9.6557*E* + 02	4.2456*E* + 02	5.5390*E* + 02
	Std	**1.0968E** − **03**	5.9258*E* − 03	3.3080*E* + 00	3.1583*E* + 01	3.6526*E* + 01	5.7272*E* + 00	4.5437*E* + 01

F32	Mean	**2.0000E** + **02**	**2.0000E** + **02**	2.7324*E* + 02	5.6117*E* + 02	5.2303*E* + 02	2.6458*E* + 02	3.3362*E* + 02
	Std	**0.0000E** + **00**	**1.4101E** − **11**	1.9475*E* + 01	4.0497*E* + 01	5.1939*E* + 01	8.5014*E* + 00	1.5205*E* + 01

**Table 11:** Statistical results for unimodal benchmark functions of different ELAPO (*n*=30).

Function	Algorithm	Min.	Mean	Max.	Std
F1	ELAPO	**4.3947E** − **207**	**7.5637E** − **200**	**7.5041E** − **199**	**0.0000E** + **00**
	ELAPO1	1.0983*E* − 196	2.0585*E* − 192	1.1337*E* − 191	**0.0000E** + **00**
	ELAPO2	1.3264*E* − 45	9.8461*E* − 45	4.1225*E* − 44	1.2223*E* − 44

F2	ELAPO	**6.6667E** − **01**	**6.6667E** − **01**	**6.6667E** − **01**	1.1703*E* − 16
	ELAPO1	**6.6667E** − **01**	**6.6667E** − **01**	**6.6667E** − **01**	**9.7912E** − **17**
	ELAPO2	**6.6667E** − **01**	**6.6667E** − **01**	**6.6667E** − **01**	5.5299*E* − 14

F3	ELAPO	−**1.0000E** + **00**	−**1.0000E** + **00**	−**1.0000E** + **00**	**0.0000E** + **00**
	ELAPO1	−**1.0000E** + **00**	−**1.0000E** + **00**	−**1.0000E** + **00**	**0.0000E** + **00**
	ELAPO2	−**1.0000E** + **00**	−**1.0000E** + **00**	−**1.0000E** + **00**	**0.0000E** + **00**

F4	ELAPO	**1.0516E** − **186**	**1.8129E** − **180**	**1.0021E** − **179**	**0.0000E** + **00**
	ELAPO1	3.4388*E* − 184	1.3329*E* − 178	6.4548*E* − 178	**0.0000E** + **00**
	ELAPO2	1.5771*E* − 42	1.5959*E* − 29	1.5959*E* − 28	5.0466*E* − 29

F5	ELAPO	**1.4582E** − **05**	**8.6010E** − **05**	**2.4099E** − **04**	**8.9264E** − **05**
	ELAPO1	2.9369*E* − 05	1.8099*E* − 04	5.7794*E* − 04	1.6862*E* − 04
	ELAPO2	1.1022*E* − 04	2.5442*E* − 04	6.2774*E* − 04	1.5210*E* − 04

F6	ELAPO	8.4106*E* − 01	**1.8767E** + **00**	**3.5521E** + **00**	**8.1532E** − **01**
	ELAPO1	9.7267*E* + 00	1.2179*E* + 01	1.3300*E* + 01	1.0108*E* + 00
	ELAPO2	**3.4946E** − **02**	2.7249*E* + 00	4.7309*E* + 00	1.5030*E* + 00

F7	ELAPO	**3.3224E** − **193**	**2.5280E** − **188**	**1.9516E** − **187**	**0.0000E** + **00**
	ELAPO1	1.8360*E* − 187	1.6201*E* − 184	9.7195*E* − 184	**0.0000E** + **00**
	ELAPO2	2.7107*E* − 42	3.5402*E* − 41	1.3260*E* − 40	3.8879*E* − 41

F8	ELAPO	**2.1127E** − **84**	**5.2137E** − **83**	**2.6909E** − **82**	**8.6122E** − **83**
	ELAPO1	1.1547*E* − 83	2.3096*E* − 81	1.0418*E* − 80	3.6979*E* − 81
	ELAPO2	2.2641*E* − 15	4.7375*E* − 15	7.9828*E* − 15	2.0046*E* − 15

F9	ELAPO	**1.1064E** − **106**	**3.6582E** − **104**	**1.8075E** − **103**	**5.3056E** − **104**
	ELAPO1	6.8578*E* − 100	1.1707*E* − 98	4.2746*E* − 98	1.3897*E* − 98
	ELAPO2	1.8562*E* − 23	4.1160*E* − 23	1.2816*E* − 22	3.2696*E* − 23

F10	ELAPO	**5.7179E** − **200**	**8.1828E** − **195**	**5.1517E** − **194**	**0.0000E** + **00**
	ELAPO1	1.4263*E* − 190	9.8501*E* − 186	9.8128*E* − 185	**0.0000E** + **00**
	ELAPO2	3.7860*E* − 45	1.1357*E* − 43	4.8673*E* − 43	1.4768*E* − 43

F11	ELAPO	**0.0000E** + **00**	**0.0000E** + **00**	**0.0000E** + **00**	**0.0000E** + **00**
	ELAPO1	**0.0000E** + **00**	**0.0000E** + **00**	**0.0000E** + **00**	**0.0000E** + **00**
	ELAPO2	5.5187*E* − 111	1.5603*E* − 107	7.9877*E* − 107	2.6781*E* − 107

**Table 12 tab12:** Statistical results for unimodal benchmark functions of different ELAPO (*n*=100).

Function	Algorithm	Min.	Mean	Max.	Std
F1	ELAPO	**1.2060E** − **201**	**5.2007E** − **193**	**4.8628E** − **192**	**0.0000E** + **00**
	ELAPO1	9.1470*E* − 188	1.2551*E* − 185	8.4746*E* − 185	**0.0000E** + **00**
	ELAPO2	1.3352*E* − 42	7.5917*E* − 41	5.2215*E* − 40	1.5892*E* − 40

F2	ELAPO	**6.6667E** − **01**	**6.6667E** − **01**	**6.6667E** − **01**	**1.1703E** − **16**
	ELAPO1	**6.6667E** − **01**	**6.6667E** − **01**	**6.6667E** − **01**	4.6472*E* − 08
	ELAPO2	**6.6667E** − **01**	**6.6667E** − **01**	**6.6667E** − **01**	**1.1703E** − **16**

F3	ELAPO	−**1.0000E** + **00**	−**1.0000E** + **00**	−**1.0000E** + **00**	**0.0000E** + **00**
	ELAPO1	−**1.0000E** + **00**	−**1.0000E** + **00**	−**1.0000E** + **00**	**0.0000E** + **00**
	ELAPO2	−**1.0000E** + **00**	−**1.0000E** + **00**	−**1.0000E** + **00**	**0.0000E** + **00**

F4	ELAPO	**2.5939E** − **179**	**3.5068E** − **174**	**2.9799E** − **173**	**0.0000E** + **00**
	ELAPO1	2.2028*E* − 176	2.5228*E* − 171	2.4291*E* − 170	**0.0000E** + **00**
	ELAPO2	2.3879*E* − 38	2.6197*E* − 25	2.6197*E* − 24	8.2841*E* − 25

F5	ELAPO	**1.6572E** − **06**	**6.9030E** − **05**	4.1043*E* − 04	1.2101*E* − 04
	ELAPO1	4.9616*E* − 05	1.3763*E* − 04	**2.8369E** − **04**	**8.4291E** − **05**
	ELAPO2	9.1224*E* − 05	2.5213*E* − 04	5.0008*E* − 04	1.3659*E* − 04

F6	ELAPO	7.5408*E* + 01	**7.8513E** + **01**	**8.1196E** + **01**	**1.6552E** + **00**
	ELAPO1	9.2775*E* + 01	9.3408*E* + 01	9.4160*E* + 01	4.6870*E* − 01
	ELAPO2	**2.5737E** − **03**	8.2837*E* + 01	1.8433*E* + 02	4.3749*E* + 01

F7	ELAPO	**5.8216E** − **187**	**1.1968E** − **182**	**8.1321E** − **182**	**0.0000E** + **00**
	ELAPO1	3.1789*E* − 181	2.5647*E* − 178	9.2747*E* − 178	**0.0000E** + **00**
	ELAPO2	2.7609*E* − 39	2.9492*E* − 36	2.7378*E* − 35	8.5881*E* − 36

F8	ELAPO	1.2973*E* − 79	**4.0687E** − **78**	**1.3134E** − **77**	**3.8556E** − **78**
	ELAPO1	**9.2722E** − **80**	5.4812*E* − 78	2.3545*E* − 77	7.5349*E* − 78
	ELAPO2	1.1764*E* − 12	6.5701*E* − 12	2.2367*E* − 11	6.4475*E* − 12

F9	ELAPO	**5.4476E** − **100**	**1.7153E** − **97**	**8.1258E** − **97**	**2.6804E** − **97**
	ELAPO1	2.1502*E* − 97	1.1376*E* − 94	7.0599*E* − 94	2.1352*E* − 94
	ELAPO2	1.4540*E* − 22	1.1495*E* − 21	2.7186*E* − 21	9.4578*E* − 22

F10	ELAPO	**4.8377E** − **192**	**2.1712E** − **185**	**1.6637E** − **184**	**0.0000E** + **00**
	ELAPO1	1.7640*E* − 181	1.7228*E* − 178	7.7291*E* − 178	**0.0000E** + **00**
	ELAPO2	7.6665*E* − 42	2.0437*E* − 40	1.2371*E* − 39	3.8039*E* − 40

F11	ELAPO	**0.0000E** + **00**	**0.0000E** + **00**	**0.0000E** + **00**	**0.0000E** + **00**
	ELAPO1	**0.0000E** + **00**	**0.0000E** + **00**	**0.0000E** + **00**	**0.0000E** + **00**
	ELAPO2	6.2316*E* − 111	2.4731*E* − 105	1.4424*E* − 104	4.8050*E* − 105

**Table 13 tab13:** Statistical results for multimodal benchmark functions of different ELAPO (*n*=30).

Function	Algorithm	Min.	Mean	Max.	Std
F12	ELAPO	**8.8818E** − **16**	**8.8818E** − **16**	**8.8818E** − **16**	**0.0000E** + **00**
	ELAPO1	**8.8818E** − **16**	**8.8818E** − **16**	**8.8818E** − **16**	**0.0000E** + **00**
	ELAPO2	**8.8818E** − **16**	**8.8818E** − **16**	**8.8818E** − **16**	**0.0000E** + **00**

F13	ELAPO	**7.4429E** − **107**	**6.1910E** − **102**	**5.5536E** − **101**	**1.7386E** − **101**
	ELAPO1	1.4046*E* − 100	8.2536*E* − 100	1.6045*E* − 99	4.1699*E* − 100
	ELAPO2	3.4320*E* − 19	3.5435*E* − 04	1.9397*E* − 03	6.1675*E* − 04

F14	ELAPO	**7.0864E** − **50**	**1.4029E** − **48**	**5.2017E** − **48**	**1.7605E** − **48**
	ELAPO1	5.9353*E* − 47	1.4937*E* − 46	2.8483*E* − 46	6.9910*E* − 47
	ELAPO2	2.1593*E* − 10	4.4564*E* − 10	7.7516*E* − 10	1.7974*E* − 10

F15	ELAPO	9.0744*E* − 01	**1.4563E** + **00**	**2.0765E** + **00**	**4.3965E** − **01**
	ELAPO1	8.3340*E* + 00	9.5357*E* + 00	1.0784*E* + 01	8.6499*E* − 01
	ELAPO2	**3.3712E** − **01**	2.5937*E* + 00	1.0258*E* + 01	3.4500*E* + 00

F16	ELAPO	**1.5705E** − **32**	**8.4188E** − **25**	**4.2955E** − **24**	**1.5385E** − **24**
	ELAPO1	1.5184*E* − 17	7.2242*E* − 17	2.2432*E* − 16	6.2807*E* − 17
	ELAPO2	**1.5705E** − **32**	7.6947*E* − 05	7.6947*E* − 04	2.4333*E* − 04

F17	ELAPO	**0.0000E** + **00**	**0.0000E** + **00**	**0.0000E** + **00**	**0.0000E** + **00**
	ELAPO1	**0.0000E** + **00**	**0.0000E** + **00**	**0.0000E** + **00**	**0.0000E** + **00**
	ELAPO2	**0.0000E** + **00**	3.2209*E* − 02	1.0586*E* − 01	4.3686*E* − 02

F18	ELAPO	−**2.9000E** + **01**	−**2.9000E** + **01**	−**2.9000E** + **01**	**0.0000E** + **00**
	ELAPO1	−**2.9000E** + **01**	−**2.9000E** + **01**	−**2.9000E** + **01**	**0.0000E** + **00**
	ELAPO2	−2.4010*E* + 01	−2.0005*E* + 01	−9.7242*E* + 00	5.3079*E* + 00

F19	ELAPO	−4.9123*E* + 03	−**4.8710E** + **03**	−**4.8355E** + **03**	**2.4102E** + **01**
	ELAPO1	−4.9107*E* + 03	−4.7477*E* + 03	−4.3972*E* + 03	1.6214*E* + 02
	ELAPO2	−**4.9239E** + **03**	−4.8603*E* + 03	−4.7473*E* + 03	5.2864*E* + 01

F20	ELAPO	**7.1841E** − **05**	**2.0743E** − **03**	**6.5185E** − **03**	**2.6236E** − **03**
	ELAPO1	1.6150*E* − 03	1.5982*E* − 02	4.3113*E* − 02	1.2385*E* − 02
	ELAPO2	1.1119*E* − 04	3.4940*E* − 03	1.2353*E* − 02	4.8505*E* − 03

F21	ELAPO	**0.0000E** + **00**	**0.0000E** + **00**	**0.0000E** + **00**	**0.0000E** + **00**
	ELAPO1	**0.0000E** + **00**	**0.0000E** + **00**	**0.0000E** + **00**	**0.0000E** + **00**
	ELAPO2	**0.0000E** + **00**	**0.0000E** + **00**	**0.0000E** + **00**	**0.0000E** + **00**

F22	ELAPO	**0.0000E** + **00**	**0.0000E** + **00**	**0.0000E** + **00**	**0.0000E** + **00**
	ELAPO1	**0.0000E** + **00**	**0.0000E** + **00**	**0.0000E** + **00**	**0.0000E** + **00**
	ELAPO2	**0.0000E** + **00**	**0.0000E** + **00**	**0.0000E** + **00**	**0.0000E** + **00**

F23	ELAPO	**5.8304E** − **90**	**7.9899E** − **02**	**9.9873E** − **02**	4.2110*E* − 02
	ELAPO1	3.3769*E* − 85	8.9886*E* − 02	**9.9873E** − **02**	3.1583*E* − 02
	ELAPO2	9.9873*E* − 02	9.9873*E* − 02	**9.9873E** − **02**	**5.2771E** − **13**

F24	ELAPO	**0.0000E** + **00**	**0.0000E** + **00**	**0.0000E** + **00**	**0.0000E** + **00**
	ELAPO1	**0.0000E** + **00**	**0.0000E** + **00**	**0.0000E** + **00**	**0.0000E** + **00**
	ELAPO2	**0.0000E** + **00**	**0.0000E** + **00**	**0.0000E** + **00**	**0.0000E** + **00**

F25	ELAPO	3.0957*E* + 02	3.4556*E* + 02	3.9799*E* + 02	3.3722*E* + 01
	ELAPO1	3.8740*E* + 02	4.0865*E* + 02	4.1395*E* + 02	**1.1183E** + **01**
	ELAPO2	**4.0677E** + **00**	**7.4112E** + **01**	**2.4969E** + **02**	7.3468*E* + 01

**Table 14 tab14:** Statistical results for multimodal benchmark functions of different ELAPO (*n*=100).

Function	Algorithm	Min.	Mean	Max.	Std
F12	ELAPO	**8.8818E** − **16**	**8.8818E** − **16**	**8.8818E** − **16**	**0.0000E** + **00**
	ELAPO1	**8.8818E** − **16**	**8.8818E** − **16**	**8.8818E** − **16**	**0.0000E** + **00**
	ELAPO2	**8.8818E** − **16**	**8.8818E** − **16**	**8.8818E** − **16**	**0.0000E** + **00**

F13	ELAPO	**8.7095E** − **103**	**3.6083E** − **99**	**1.8257E** − **98**	**5.4747E** − **99**
	ELAPO1	2.5297*E* − 98	2.0018*E* − 96	1.4412*E* − 95	4.4005*E* − 96
	ELAPO2	4.9659*E* − 04	1.0832*E* − 02	4.9968*E* − 02	1.6131*E* − 02

F14	ELAPO	**2.0856E** − **46**	**1.7929E** − **44**	**9.4887E** − **44**	**2.8722E** − **44**
	ELAPO1	3.9777*E* − 45	4.6572*E* − 44	1.1290*E* − 43	3.4345*E* − 44
	ELAPO2	1.5153*E* − 08	7.9214*E* − 08	3.0473*E* − 07	8.5298*E* − 08

F15	ELAPO	**3.1824E** + **00**	**7.2183E** + **00**	**3.2412E** + **01**	8.8821*E* + 00
	ELAPO1	3.9427*E* + 01	4.0794*E* + 01	4.1923*E* + 01	**8.9874E** − **01**
	ELAPO2	3.3002*E* + 00	1.7828*E* + 01	4.0400*E* + 01	1.7857*E* + 01

F16	ELAPO	**4.7116E** − **33**	**4.7116E** − **33**	**4.7116E** − **33**	**1.9082E** − **48**
	ELAPO1	7.3562*E* − 05	1.4325*E* − 04	2.6188*E* − 04	5.4182*E* − 05
	ELAPO2	**4.7116E** − **33**	3.1101*E* − 03	3.1101*E* − 02	9.8349*E* − 03

F17	ELAPO	**0.0000E** + **00**	**0.0000E** + **00**	**0.0000E** + **00**	**0.0000E** + **00**
	ELAPO1	**0.0000E** + **00**	**0.0000E** + **00**	**0.0000E** + **00**	**0.0000E** + **00**
	ELAPO2	**0.0000E** + **00**	1.0827*E* − 02	5.1706*E* − 02	1.7751*E* − 02

F18	ELAPO	−**9.9000E** + **01**	−**7.4063E** + **01**	−2.6004*E* + 01	3.2165*E* + 01
	ELAPO1	−**9.9000E** + **01**	−2.3413*E* + 01	−1.2547*E* + 01	2.6599*E* + 01
	ELAPO2	−8.0137*E* + 01	−6.3474*E* + 01	−**2.8524E** + **01**	**2.0801E** + **01**

F19	ELAPO	−1.3042*E* + 05	−6.5750*E* + 04	−9.5402*E* + 02	4.1853*E* + 04
	ELAPO1	−3.0396*E* + 03	−2.5921*E* + 03	−2.1467*E* + 03	**2.2698E** + **02**
	ELAPO2	−**1.3365E** + **05**	−**7.7752E** + **04**	−**3.4716E** + **04**	3.3300*E* + 04

F20	ELAPO	**3.4881E** − **04**	**7.3630E** − **02**	**4.5528E** − **01**	**1.5569E** − **01**
	ELAPO1	8.5545*E* − 01	1.6562*E* + 00	2.0958*E* + 00	3.8738*E* − 01
	ELAPO2	9.3205*E* − 04	1.2621*E* − 01	5.5200*E* − 01	1.7669*E* − 01

F21	ELAPO	**0.0000E** + **00**	**0.0000E** + **00**	**0.0000E** + **00**	**0.0000E** + **00**
	ELAPO1	**0.0000E** + **00**	**0.0000E** + **00**	**0.0000E** + **00**	**0.0000E** + **00**
	ELAPO2	**0.0000E** + **00**	**0.0000E** + **00**	**0.0000E** + **00**	**0.0000E** + **00**

F22	ELAPO	**0.0000E** + **00**	**0.0000E** + **00**	**0.0000E** + **00**	**0.0000E** + **00**
	ELAPO1	**0.0000E** + **00**	**0.0000E** + **00**	**0.0000E** + **00**	**0.0000E** + **00**
	ELAPO2	**0.0000E** + **00**	**0.0000E** + **00**	**0.0000E** + **00**	**0.0000E** + **00**

F23	ELAPO	**9.9873E** − **02**	**9.9873E** − **02**	**9.9873E** − **02**	**7.7820E** − **17**
	ELAPO1	**9.9873E** − **02**	**9.9873E** − **02**	**9.9873E** − **02**	9.4568*E* − 11
	ELAPO2	**9.9873E** − **02**	**9.9873E** − **02**	**9.9873E** − **02**	9.6023*E* − 16

F24	ELAPO	**0.0000E** + **00**	**0.0000E** + **00**	**0.0000E** + **00**	**0.0000E** + **00**
	ELAPO1	**0.0000E** + **00**	**0.0000E** + **00**	**0.0000E** + **00**	**0.0000E** + **00**
	ELAPO2	**0.0000E** + **00**	**0.0000E** + **00**	**0.0000E** + **00**	**0.0000E** + **00**

F25	ELAPO	1.5069*E* + 03	3.6192*E* + 03	4.2156*E* + 03	8.1059*E* + 02
	ELAPO1	4.5995*E* + 03	4.5995*E* + 03	4.5995*E* + 03	**9.5869E** − **13**
	ELAPO2	**2.3949E** + **01**	**1.0427E** + **03**	**3.1627E** + **03**	1.1139*E* + 03

**Table 15 tab15:** Statistical results for CEC 2014 benchmark functions of different ELAPO (*n*=30).

Function	Algorithm	Min.	Mean	Max.	Std
F26	ELAPO	1.7031*E* + 05	**4.4635E** + **05**	**1.4637E** + **06**	**4.1890E** + **05**
	ELAPO1	1.3255*E* + 05	7.8737*E* + 05	1.6349*E* + 06	5.4983*E* + 05
	ELAPO2	**8.6131E** + **04**	6.9261*E* + 05	2.3688*E* + 06	6.8875*E* + 05

F27	ELAPO	2.0002*E* + 02	**2.0030E** + **02**	**2.0173E** + **02**	**5.2289E** − **01**
	ELAPO1	2.1032*E* + 02	4.5935*E* + 02	9.2097*E* + 02	2.0827*E* + 02
	ELAPO2	**2.0000E** + **02**	2.0045*E* + 02	2.0248*E* + 02	7.6958*E* − 01

F28	ELAPO	**4.0001E** + **02**	**4.0704E** + **02**	**4.6723E** + **02**	2.1148*E* + 01
	ELAPO1	4.0408*E* + 02	4.6828*E* + 02	5.6357*E* + 02	4.7798*E* + 01
	ELAPO2	**4.0001E** + **02**	4.0766*E* + 02	4.6765*E* + 02	**2.1129E** + **01**

F29	ELAPO	1.3442*E* + 04	**1.6246E** + **05**	**3.3345E** + **05**	**1.0464E** + **05**
	ELAPO1	1.0292*E* + 05	2.6555*E* + 05	6.1334*E* + 05	2.0477*E* + 05
	ELAPO2	**6.9968E** + **03**	4.2214*E* + 05	1.5059*E* + 06	5.2014*E* + 05

F30	ELAPO	**2.6152E** + **03**	**2.6152E** + **03**	**2.6152E** + **03**	**4.7935E** − **13**
	ELAPO1	**2.6152E** + **03**	**2.6152E** + **03**	**2.6152E** + **03**	1.2234*E* − 11
	ELAPO2	**2.6152E** + **03**	**2.6152E** + **03**	**2.6152E** + **03**	3.6190*E* − 12

F31	ELAPO	**2.6000E** + **03**	**2.6000E** + **03**	**2.6000E** + **03**	3.5666*E* − 04
	ELAPO1	**2.6000E** + **03**	**2.6000E** + **03**	**2.6000E** + **03**	**1.7811E** − **04**
	ELAPO2	**2.6000E** + **03**	**2.6000E** + **03**	**2.6000E** + **03**	1.5511*E* − 03

F32	ELAPO	**2.7000E** + **03**	**2.7000E** + **03**	**2.7000E** + **03**	**0.0000E** + **00**
	ELAPO1	**2.7000E** + **03**	**2.7000E** + **03**	**2.7000E** + **03**	**0.0000E** + **00**
	ELAPO2	**2.7000E** + **03**	**2.7000E** + **03**	**2.7000E** + **03**	**0.0000E** + **00**

**Table 16:** Statistical results for CEC 2014 benchmark functions of different ELAPO (*n*=100).

Function	Algorithm	Min.	Mean	Max.	Std
F26	ELAPO	2.4634*E* + 06	**5.2161E** + **06**	**1.3141E** + **07**	**3.1061E** + **06**
	ELAPO1	2.2387*E* + 07	4.5695*E* + 07	6.2258*E* + 07	1.4119*E* + 07
	ELAPO2	**2.4614E** + **06**	7.0362*E* + 06	1.5330*E* + 07	4.6938*E* + 06

F27	ELAPO	**6.2568E** + **02**	**7.8528E** + **03**	**1.6154E** + **04**	**4.6668E** + **03**
	ELAPO1	9.6085*E* + 07	2.1771*E* + 08	3.3325*E* + 08	6.8438*E* + 07
	ELAPO2	1.4145*E* + 03	1.0199*E* + 04	2.4210*E* + 04	7.4670*E* + 03

F28	ELAPO	4.8012*E* + 02	**5.4633E** + **02**	**6.3713E** + **02**	**4.9530E** + **01**
	ELAPO1	7.2188*E* + 02	8.8310*E* + 02	9.4605*E* + 02	7.5816*E* + 01
	ELAPO2	**4.0910E** + **02**	5.6801*E* + 02	6.3932*E* + 02	6.6571*E* + 01

F29	ELAPO	8.6796*E* + 05	**2.0363E** + **06**	**5.4231E** + **06**	**1.2767E** + **06**
	ELAPO1	1.2845*E* + 06	3.6401*E* + 06	7.6576*E* + 06	2.5212*E* + 06
	ELAPO2	**7.0505E** + **05**	2.1188*E* + 06	6.4199*E* + 06	1.7726*E* + 06

F30	ELAPO	**2.5000E** + **03**	2.6451*E* + 03	2.7624*E* + 03	**6.2281E** + **01**
	ELAPO1	**2.5000E** + **03**	2.7849*E* + 03	3.4244*E* + 03	3.5275*E* + 02
	ELAPO2	**2.5000E** + **03**	**2.6186E** + **03**	**2.6483E** + **03**	6.2509*E* + 01

F31	ELAPO	**2.6000E** + **03**	**2.6000E** + **03**	**2.6000E** + **03**	1.0968*E* − 03
	ELAPO1	**2.6000E** + **03**	**2.6000E** + **03**	**2.6000E** + **03**	**6.0886E** − **04**
	ELAPO2	**2.6000E** + **03**	**2.6000E** + **03**	**2.6000E** + **03**	4.5087*E* − 03

F32	ELAPO	**2.7000E** + **03**	**2.7000E** + **03**	**2.7000E** + **03**	**0.0000E** + **00**
	ELAPO1	**2.7000E** + **03**	**2.7000E** + **03**	**2.7000E** + **03**	**0.0000E** + **00**
	ELAPO2	**2.7000E** + **03**	**2.7000E** + **03**	**2.7000E** + **03**	**0.0000E** + **00**

**Table 17:** Results of Wilcoxon's test for ELAPO against other six algorithms for each benchmark function with 10 independent runs at *n*=30 (*α* = 0.05).

Function	LAPO vs. ELAPO		SSA vs. ELAPO		Jaya vs. ELAPO		IBB-BC vs. ELAPO		ODE1 vs. ELAPO		ALO vs. ELAPO	
	*p* value	Win	*p* value	Win	*p* value	Win	*p* value	Win	*p* value	Win	*p* value	Win
F1	6.5157*E* − 02	−	3.8030*E* − 05	**+**	8.8057*E* − 06	**+**	1.8054*E* − 02	**+**	3.3181*E* − 01	−	1.0788*E* − 01	−
F2	1.1430*E* − 03	**+**	3.5370*E* − 01	−	8.2032*E* − 02	−	2.0905*E* − 01	−	1.6587*E* − 01	−	4.1164*E* − 02	**+**
F3	—	**=**	1.4851*E* − 04	**+**	4.8142*E* − 06	**+**	2.1981*E* − 17	**+**	1.4807*E* − 02	**+**	8.7627*E* − 05	**+**
F4	2.3713*E* − 01	−	6.8108*E* − 03	**+**	1.2111*E* − 05	**+**	1.1967*E* − 04	**+**	1.3930*E* − 01	−	1.6670*E* − 04	**+**
F5	3.2636*E* − 03	**+**	1.1738*E* − 06	**+**	2.1936*E* − 09	**+**	6.6659*E* − 09	**+**	6.8124*E* − 07	**+**	1.3194*E* − 05	**+**
F6	3.9780*E* − 11	**+**	5.6264*E* − 04	**+**	1.4458*E* − 04	**+**	4.7296*E* − 02	**+**	3.8957*E* − 04	**+**	1.3327*E* − 02	**+**
F7	1.3127*E* − 02	**+**	4.2515*E* − 04	**+**	2.6918*E* − 05	**+**	4.0856*E* − 02	**+**	2.0166*E* − 01	−	2.4269*E* − 02	**+**
F8	1.0554*E* − 03	**+**	2.6806*E* − 07	**+**	1.3055*E* − 04	**+**	3.2266*E* − 03	**+**	6.0020*E* − 05	**+**	1.4067*E* − 06	**+**
F9	1.7774*E* − 01	−	1.7559*E* − 06	**+**	3.1821*E* − 06	**+**	4.0316*E* − 04	**+**	1.8318*E* − 05	**+**	4.8798*E* − 02	**+**
F10	6.0410*E* − 02	−	4.0552*E* − 03	**+**	3.8508*E* − 06	**+**	3.2237*E* − 05	**+**	1.1459*E* − 02	**+**	4.8526*E* − 03	**+**
F11	2.5412*E* − 01	−	2.3083*E* − 01	−	1.8567*E* − 01	−	1.6998*E* − 02	**+**	2.2426*E* − 01	−	9.9079*E* − 04	**+**
F12	5.7296*E* − 03	**+**	1.1192*E* − 02	**+**	4.6725*E* − 03	**+**	3.7373*E* − 14	**+**	3.2594*E* − 01	−	8.0055*E* − 05	**+**
F13	1.8191*E* − 03	**+**	1.8814*E* − 04	**+**	8.9399*E* − 05	**+**	2.5553*E* − 03	**+**	1.8822*E* − 01	−	5.4968*E* − 04	**+**
F14	9.5904*E* − 04	**+**	8.1454*E* − 07	**+**	4.6052*E* − 08	**+**	6.4579*E* − 09	**+**	6.2990*E* − 07	**+**	6.7484*E* − 09	**+**
F15	1.3848*E* − 10	**+**	1.2732*E* − 03	**+**	3.4198*E* − 13	**+**	3.2496*E* − 12	**+**	1.9113*E* − 13	**+**	7.5973*E* − 11	**+**
F16	3.4344*E* − 01	−	1.3527*E* − 03	**+**	1.9997*E* − 05	**+**	3.6772*E* − 02	**+**	2.9349*E* − 02	**+**	1.0442*E* − 05	**+**
F17	3.4344*E* − 01	−	6.7604*E* − 04	**+**	2.1918*E* − 03	**+**	4.1350*E* − 02	**+**	2.0726*E* − 02	**+**	1.0132*E* − 02	**+**
F18	1.3100*E* − 05	**+**	1.2328*E* − 06	**+**	3.2377*E* − 14	**+**	2.2121*E* − 17	**+**	7.6070*E* − 14	**+**	3.5169*E* − 09	**+**
F19	2.9586*E* − 04	**+**	3.4911*E* − 03	**+**	7.4745*E* − 02	−	9.9030*E* − 03	**+**	1.7725*E* − 05	**+**	7.2303*E* − 06	**+**
F20	2.8427*E* − 02	**+**	6.2178*E* − 02	**+**	4.0529*E* − 05	**+**	1.7734*E* − 01	−	1.1848*E* − 01	−	8.2189*E* − 04	**+**
F21	—	**=**	7.0957*E* − 02	**+**	6.6615*E* − 11	**+**	4.8172*E* − 06	**+**	1.5624*E* − 08	**+**	4.3379*E* − 06	**+**
F22	1.6787*E* − 01	−	8.1058*E* − 02	**+**	6.4191*E* − 12	**+**	1.2747*E* − 06	**+**	7.1310*E* − 06	**+**	4.4267*E* − 04	**+**
F23	1.6785*E* − 01	−	3.4082*E* − 10	**+**	5.7993*E* − 11	**+**	3.9727*E* − 07	**+**	1.0620*E* − 05	**+**	2.6960*E* − 08	**+**
F24	—	**=**	2.9637*E* − 05	**+**	5.0207*E* − 05	**+**	1.7982*E* − 06	**+**	1.6055*E* − 01	−	4.2089*E* − 08	**+**
F25	2.7440*E* − 04	**+**	6.8708*E* − 01	−	9.8070*E* − 12	**+**	9.7584*E* − 12	**+**	2.6606*E* − 08	**+**	1.6495*E* − 09	**+**
F26	6.1439*E* − 02	−	7.0291*E* − 04	**+**	1.3762*E* − 05	**+**	3.8405*E* − 03	**+**	6.1551*E* − 03	**+**	5.0196*E* − 05	**+**
F27	6.8071*E* − 03	**+**	7.0180*E* − 03	**+**	1.5724*E* − 09	**+**	1.7808*E* − 04	**+**	5.2722*E* − 02	−	5.0467*E* − 04	**+**
F28	3.6344*E* − 03	**+**	4.8441*E* − 05	**+**	3.0928*E* − 08	**+**	3.2500*E* − 09	**+**	1.7440*E* − 07	**+**	1.9483*E* − 05	**+**
F29	4.6908*E* − 02	**+**	4.9702*E* − 04	**+**	1.1193*E* − 04	**+**	1.0340*E* − 02	**+**	3.2293*E* − 03	**+**	4.3079*E* − 03	**+**
F30	2.5163*E* − 02	**+**	2.6125*E* − 02	**+**	2.8404*E* − 07	**+**	3.5374*E* − 05	**+**	3.2647*E* − 01	−	1.3533*E* − 04	**+**
F31	2.6889*E* − 07	**+**	4.1868*E* − 07	**+**	3.8867*E* − 09	**+**	2.0918*E* − 10	**+**	4.8250*E* − 08	**+**	2.7003*E* − 10	**+**
F32	1.6785*E* − 01	−	6.3084*E* − 04	**+**	1.5043*E* − 09	**+**	4.1352*E* − 07	**+**	2.5620*E* − 10	**+**	8.4991*E* − 08	**+**
+/−	—	18/11	—	26/6	—	29/3		30/2		21/11		31/1

**Table 18:** Results of Wilcoxon's test for SSA against other six algorithms for each benchmark function with 10 independent runs at *n* = 100 (*α* = 0.05).

Function	LAPO vs. ELAPO		SSA vs. ELAPO		Jaya vs. ELAPO		IBB-BC vs. ELAPO		ODE1 vs. ELAPO		ALO vs. ELAPO	
	*p* value	Win	*p* value	Win	*p* value	Win	*p* value	Win	*p* value	Win	*p* value	Win
F1	1.5770*E* − 01	−	4.2350*E* − 08	**+**	1.5231*E* − 07	**+**	6.3386*E* − 04	**+**	1.3419*E* − 03	**+**	3.5682*E* − 06	**+**
F2	7.6218*E* − 03	**+**	6.0367*E* − 08	**+**	1.9065*E* − 04	**+**	9.3693*E* − 03	**+**	3.2161*E* − 05	**+**	5.1856*E* − 07	**+**
F3	—	**=**	3.5796*E* − 08	**+**	2.2802*E* − 07	**+**	4.4140*E* − 54	**+**	2.3346*E* − 02	**+**	3.0033*E* − 05	**+**
F4	1.3041*E* − 01	−	3.5293*E* − 08	**+**	2.8240*E* − 04	**+**	1.4775*E* − 06	**+**	2.7093*E* − 02	**+**	9.4948*E* − 07	**+**
F5	5.6333*E* − 03	**+**	1.0084*E* − 08	**+**	4.8518*E* − 06	**+**	1.3531*E* − 06	**+**	9.5032*E* − 05	**+**	6.7646*E* − 10	**+**
F6	9.5047*E* − 12	**+**	3.3028*E* − 06	**+**	1.8052*E* − 05	**+**	2.2892*E* − 03	**+**	2.4625*E* − 02	**+**	8.5749*E* − 03	**+**
F7	1.4156*E* − 01	−	3.0753*E* − 08	**+**	6.9812*E* − 08	**+**	3.8161*E* − 04	**+**	2.0774*E* − 03	**+**	1.8206*E* − 06	**+**
F8	1.9728*E* − 04	**+**	1.3132*E* − 12	**+**	5.7059*E* − 11	**+**	5.6270*E* − 11	**+**	2.1460*E* − 08	**+**	2.7677*E* − 09	**+**
F9	2.7143*E* − 02	**+**	4.6832*E* − 11	**+**	3.2634*E* − 07	**+**	2.7294*E* − 10	**+**	5.0914*E* − 05	**+**	2.2988*E* − 03	**+**
F10	4.3062*E* − 02	**+**	4.2546*E* − 08	**+**	1.6382*E* − 06	**+**	8.1021*E* − 03	**+**	9.3687*E* − 02	−	1.0917*E* − 04	**+**
F11	2.6996*E* − 01	−	2.2696*E* − 01	−	5.0667*E* − 02	−	1.1055*E* − 03	**+**	3.3891*E* − 01	−	1.2744*E* − 03	**+**
F12	7.6477*E* − 02	−	5.8330*E* − 05	**+**	3.0288*E* − 11	**+**	4.6170*E* − 19	**+**	8.3106*E* − 07	**+**	6.0946*E* − 08	**+**
F13	1.0360*E* − 03	**+**	5.5028*E* − 07	**+**	9.0010*E* − 09	**+**	5.9879*E* − 09	**+**	1.9392*E* − 03	**+**	2.4309*E* − 06	**+**
F14	3.8838*E* − 04	**+**	9.7111*E* − 10	**+**	1.2974*E* − 11	**+**	9.7901*E* − 14	**+**	4.5811*E* − 09	**+**	3.3538*E* − 11	**+**
F15	8.3808*E* − 07	**+**	9.8135*E* − 05	**+**	2.3964*E* − 07	**+**	3.5313*E* − 07	**+**	1.6716*E* − 07	**+**	1.0160*E* − 06	**+**
F16	3.1494*E* − 01	−	1.4591*E* − 05	**+**	9.7572*E* − 02	−	1.4803*E* − 03	**+**	8.8968*E* − 02	−	5.9325*E* − 06	**+**
F17	—	**=**	1.1073*E* − 09	**+**	4.1210*E* − 12	**+**	1.6710*E* − 08	**+**	2.9375*E* − 05	**+**	1.7779*E* − 07	**+**
F18	3.9961*E* − 04	**+**	6.4253*E* − 01	−	1.7095*E* − 04	**+**	8.8954*E* − 05	**+**	2.0868*E* − 04	**+**	1.9220*E* − 03	**+**
F19	9.8149*E* − 04	**+**	1.1788*E* − 06	**+**	4.8892*E* − 07	**+**	2.1151*E* − 05	**+**	2.0236*E* − 06	**+**	1.0299*E* − 02	**+**
F20	4.3629*E* − 07	**+**	5.7602*E* − 01	−	6.2070*E* − 09	**+**	1.1723*E* − 02	**+**	6.6614*E* − 02	−	2.9847*E* − 04	**+**
F21	—	**=**	3.5828*E* − 11	**+**	2.7658*E* − 12	**+**	7.1919*E* − 11	**+**	7.3339*E* − 10	**+**	1.1238*E* − 07	**+**
F22	—	**=**	7.0366*E* − 09	**+**	3.4023*E* − 12	**+**	3.3868*E* − 10	**+**	2.6364*E* − 12	**+**	7.8276*E* − 07	**+**
F23	5.2427*E* − 02	−	2.3972*E* − 10	**+**	1.0601*E* − 11	**+**	2.3959*E* − 12	**+**	4.1327*E* − 09	**+**	4.4496*E* − 09	**+**
F24	—	**=**	1.4813*E* − 09	**+**	1.4883*E* − 11	**+**	2.4103*E* − 13	**+**	1.3202*E* − 08	**+**	1.0014*E* − 11	**+**
F25	4.0643*E* − 03	**+**	1.3414*E* − 09	**+**	1.9271*E* − 04	**+**	3.5879*E* − 09	**+**	1.1709*E* − 02	**+**	6.5374*E* − 10	**+**
F26	9.1228*E* − 07	**+**	2.4299*E* − 06	**+**	4.8229*E* − 08	**+**	2.4166*E* − 07	**+**	2.9383*E* − 05	**+**	9.1708*E* − 07	**+**
F27	1.9144*E* − 02	**+**	8.2124*E* − 08	**+**	3.2662*E* − 10	**+**	2.7758*E* − 04	**+**	6.3573*E* − 03	**+**	9.5400*E* − 04	**+**
F28	2.7429*E* − 07	**+**	2.3276*E* − 07	**+**	1.2105*E* − 07	**+**	1.2547*E* − 07	**+**	1.7003*E* − 06	**+**	9.3832*E* − 09	**+**
F29	5.8224*E* − 02	−	1.1899*E* − 03	**+**	1.0274*E* − 09	**+**	2.5110*E* − 03	**+**	2.2029*E* − 05	**+**	4.0114*E* − 04	**+**
F30	7.7749*E* − 01	−	7.1957*E* − 01	−	2.2963*E* − 07	**+**	6.5996*E* − 07	**+**	7.2799*E* − 01	−	1.1532*E* − 04	**+**
F31	2.3273*E* − 05	**+**	3.4931*E* − 17	**+**	4.5281*E* − 12	**+**	2.0453*E* − 13	**+**	7.3367*E* − 16	**+**	1.4373*E* − 09	**+**
F32	1.0442*E* − 02	**+**	8.3154*E* − 07	**+**	4.3080*E* − 10	**+**	1.0525*E* − 08	**+**	1.7940*E* − 09	**+**	4.9109*E* − 10	**+**
+/−	—	18/9	—	28/4	—	30/2	—	32/0	—	27/5	—	32/0

**Table 19:** Friedman ranks for each benchmark function of all algorithms at *n*=30.

Fun	ELAPO	LAPO	SSA	Jaya	IBB-BC	ODE1	ALO
F1	1	2	4	5	6	3	7
F2	1.5	1.5	4	5	7	3	6
F3	2	2	5	6	7	2	4
F4	1	2	4	5	7	3	6
F5	1	2	7	6	3	4	5
F6	1	2	4	5	7	3	6
F7	1	2	4	5	6	3	7
F8	1	2	4	5	3	6	7
F9	1	2	4	6	5	3	7
F10	1	2	5	7	6	3	4
F11	1	2	3	4	6	5	7
F12	1	2	4	5	7	3	6
F13	1	2	3	7	5	4	6
F14	1	2	4	5	6	3	7
F15	2	3	1	6	7	5	4
F16	1	3	2	6	4	5	7
F17	1	2	5	7	6	3	4
F18	1	3	2	6	7	5	4
F19	1	2	6	4	7	5	3
F20	2	6	1	7	3	4	5
F21	1.5	1.5	3	7	4	6	5
F22	1	3	2	7	4	6	5
F23	1	2	5	6	7	3	4
F24	1.5	1.5	4	5	6	3	7
F25	1	3	2	6	7	5	4
F26	1	2	4	7	5	3	6
F27	1	2	6	7	4	3	5
F28	1	2	4	7	6	3	5
F29	2	3	6	7	4	1	5
F30	2	2	2	6	7	3	5
F31	1.5	1.5	4	6	7	3	5
F32	1.5	1.5	4	6	7	3	5
Average	1.234375	2.234375	3.8125	5.90625	5.71875	3.65625	5.40625
Rank	1	2	4	7	6	3	5

**Table 20:** Friedman ranks for each benchmark function of all algorithms at *n*=100.

Fun	ELAPO	LAPO	SSA	Jaya	IBB-BC	ODE1	ALO
F1	1	2	3	6	7	4	5
F2	1.5	1.5	5	7	3	6	4
F3	2	2	4	6	7	5	2
F4	1	2	3	5	7	4	6
F5	1	2	6	7	3	4	5
F6	1	2	5	7	3	6	4
F7	1	2	3	6	7	4	5
F8	1	2	7	5	6	3	4
F9	1	2	4	5	6	3	7
F10	1	2	4	7	6	5	3
F11	1	2	3	7	5	4	6
F12	1	2	6	5	7	3	4
F13	1	2	4	7	6	3	5
F14	1	2	4	6	7	3	5
F15	1	3	2	7	5	6	4
F16	1	2	4	7	3	6	5
F17	1.5	1.5	5	6	7	4	3
F18	1	4	2	6	7	5	3
F19	1	3	6	5	7	4	2
F20	2	6	1	7	4	3	5
F21	1.5	1.5	3	7	5	6	4
F22	1.5	1.5	3	7	5	6	4
F23	1.5	1.5	3	6	7	4	5
F24	1.5	1.5	4	5	7	3	6
F25	1	2	4	7	3	6	5
F26	1	2	5	7	6	3	4
F27	1	5	2	7	6	4	3
F28	1	4	2	7	6	3	5
F29	1	2	6	7	4	5	3
F30	1	2	4	7	6	3	5
F31	1.5	1.5	3	6	7	4	5
F32	1.5	1.5	4	7	6	3	5
Average	1.1875	2.28125	3.875	6.375	5.65625	4.21875	4.40625
Rank	1	2	3	7	6	4	5

**Table 21:** Ranking of algorithms using MAE.

Algorithm	MAE (*n*=30)	Rank	MAE (*n*=100)	Rank
ELAPO	1.9004*E* + 04	1	2.3027*E* + 05	1
LAPO	3.6355*E* + 04	2	2.8935*E* + 05	2
ODE1	7.8336*E* + 04	3	1.3912*E* + 07	5
SSA	2.7223*E* + 05	4	1.0381*E* + 07	3
IBB-BC	4.4013*E* + 05	5	9.3375*E* + 08	6
ALO	4.7895*E* + 05	6	1.3480*E* + 07	4
Jaya	2.1949*E* + 08	7	4.1882*E* + 09	7

## Data Availability

The data used to support the findings of this study are available from the corresponding author upon request.
